# Gender in suicide prevention in adolescents and young adults: a systematic review

**DOI:** 10.1186/s12889-026-28223-1

**Published:** 2026-07-09

**Authors:** Léa Loubet, Virginie Bonnot, Joëlle Kivits

**Affiliations:** 1 Université Paris Cité, Inserm, ECEVE, Paris, F-75010 France; 2 Laboratoire de Psychologie Sociale, Université Paris Cité, Boulogne- Billancourt, F-92100 France

**Keywords:** Suicide, Suicidal behaviour, Adolescent, Young adult, Intervention, Prevention, Gender

## Abstract

**Background:**

Suicidal behaviour in adolescents and young adults (AYA) continues to be a major global concern. The concept of gender has emerged as a pivotal element in public health and for research institutions. The objective was to assess the current use of a gendered approach in suicide prevention for AYA.

**Method:**

To that aim we conducted a systematic review of suicidal behaviour prevention programs and mobilised the gender transformative framework for health promotion developed by Pederson et al. [1].

**Results:**

The 133 included papers were distributed along a continuum with the majority of them being gender-blind (49%). A noteworthy proportion of the papers could be categorised as gender sensitive at varying degrees (41%). The gender specific studies (8%) had the most gender-related content, with three studies incorporating gender transformative elements (2%). The study reveals that the integration of gender remains limited, with a predominant focus on the differences between young women and men. The gender studies are not included in the theoretical basis of the interventions.

**Conclusions:**

Therefore, we argue that integrating a gender perspective necessitates the incorporation of novel bodies of literature and theoretical discussion, as evidenced in critical suicide studies. Consequently, the content of the preventive programs and the professional positioning in light of gender and intersectional frameworks represent a subject of particular interest. The integration of a multifaceted gender definition within the evaluation design could prove advantageous. In the end, the gender transformative method provides some interesting elements to incorporate new frameworks for suicide in future prevention research.

**Supplementary Information:**

The online version contains supplementary material available at https://doi.org/10.1186/s12889-026-28223-1.

## Introduction

In France, the use of emergency services due to suicide ideation and self-injury has shown a striking rise since 2020 among individuals aged 10 to 24 and particularly young women [2]. Suicide remains one of the leading preventable causes of death among adolescents and young adults (AYA) [3]. Regarding gender, statistical data on suicidal behaviour reveal differences in prevalence, risk, and protective factors between sex/gender identity categories [4]. Young women present a higher risk of suicidal behaviour while young men have a higher risk of death by suicide [4]. Additionally, the expression of distress can be influenced by social constructs and socialisation, which may be gendered [5]. This includes suicide scripts, method choices, and help-seeking behaviour [6]. Studies have shown that gender socialisation during adolescence encompasses harmful gender norms in a variety of contexts [7, 8] and that gender affects suicidal behaviour in this population [4, 9–15]. The acknowledgment of gender norms could be particularly beneficial for programs designed for this specific demographic population [16]. Therefore, the present paper focuses on this developmental period as a potential opportunity for intervention.

From the perspectives of feminist materialism and sociology, gender can be conceptualised as a social system that determines the position of individuals and their access to resources and power [17–19]. In health research, this gender system refers to a set of expectations, roles, and norms that are associated with the binary distinction of human beings based on the sex assigned at birth [19–22]. The assignment of sex at birth establishes a sex category that is often used as a proxy for gender identity, despite its inherent limitations [23]. Moreover, the conceptualisation of sex as a binary feature continues to be a subject of extensive discussion in the literature [24, 25]. Consequently, a gender approach entails a multifaceted approach, and the consideration of gender through four dimensions: gender identity, gender roles, gender relations and institutionalised gender [22]^1^.

Along with young women, sexual and gender minority youth are also considered to be at higher risk for suicidal behaviour [26, 27], in part because LGBTI+ individuals challenge cisgender and heteronormative gender norms and relations and because of the structures that perpetuate discrimination [28, 29]. As such, the concepts of gender and sexual orientation are distinct yet interrelated. In particular, the intersection of sexism and heteronormativity has been demonstrated to perpetuate gender conformity and systematic inequalities [30–32]. More generally the distress experienced by young people is associated with various social positions and structures of oppression, as evidenced among non-white sexual minority young people [33, 34]. Accordingly, scholars have advocated for the integration of an intersectional framework into suicide studies [33, 35]. It is therefore essential to consider the experiences of gender and sexual minorities, as well as other significant social positions and oppressive structures when reflecting on the integration of gender [36].

Thereby, Pederson et al. developed a framework on gender transformative health promotion [1]. This framework delineates a continuum that ranges from gender-blindness to gender transformative health promotion [1, 22]. Gender-blind programs ignore gender in research questions and analysis, as well as the potential effects of gender norms, roles and relations on health outcomes [22]. Gender sensitive programs are characterised by their acknowledgement of gender-based differences in health issues, for instance in the rationale and research questions, and they include gender constructs into measures and evaluation, but do not introduce these elements further into action [22]. Gender specific programs are designed to address the unique needs of a particular group from the research design to the program content [22]. Finally, gender transformative programs are designed to address health issues and inequalities by challenging and transforming gender norms and interactions in conjunction with other social characteristics such as race/ethnicity or class [22]. These elements are addressed from the research question to the methods employed, the content of the program, the evaluation design, and the analysis [22]. The gender transformative framework has already been utilised in the context of sexual health programs and has proven useful in addressing harmful norms of masculinity [37] or relationships and violence in sexual health interventions [38]. To the best of our knowledge, this topic has not yet been studied or integrated into research on suicide prevention intervention research, except in a narrative review [39] despite its potential value, particularly in light of the literature linking gender and suicide. Although there is a lack of empirical studies on the utility of a gender lens in suicide prevention strategies, the extant literature on the use of gendered approaches in public health prompts us to consider its integration into our methodologies [22, 38, 40–42]. In addition, the *Lancet* Commission on Gender and Global Health has recently reminded that gender is a pivotal factor in achieving social justice and equity [43].

Previous systematic reviews on suicidal behaviour prevention have highlighted the diversity of therapeutic programs [44], the varying degrees of the effectiveness of psychosocial interventions [45], the increase in the number of intervention studies, or their fluctuating effects depending on the settings [14, 46]. Suicidology is a complex field and has been criticised for the predominance of pathological and individualistic frameworks in the understanding suicidal behaviour [47]. This emphasis may constrain interventions to medicalised, individual-level strategies, potentially overlooking broader structural determinants [47, 48]. Consequently, there is a growing recognition of the need for interdisciplinary approaches and innovative models of care [49, 50]. By drawing on a gender transformative framework, the present work adopts a critical perspective on prevailing models of suicide prevention intervention and seek to contribute to the development and operationalisation of new approaches to prevention.

Regarding gender, two systematic review noted that the programs were evaluated using mostly samples of young women [14, 45] which could limit the assessment of suicide prevention effectiveness in young men [14]. Moreover, Robinson et al. emphasised the lack of interventions targeting specific groups, including sexual and gender minority youth [14]. However, to date, none of the reviews have systematically investigated whether and how gender is problematized in suicide prevention.

Given the Pederson et al. framework [1], the purpose of this systematic review was to describe the current use of a gendered approach in AYA suicide prevention as follows: (1) to highlight how gender data are considered and translated when developing and implementing suicide prevention interventions and (2) to identify and describe gender transformative approaches in suicide prevention programs. As such, the objective was not to evaluate the efficacy of the interventions, but rather to examine the use of a gender lens, as a preliminary step to consider gendered approaches.

## Methods

The current study has followed the preferred reporting items for systematic review and meta-analysis (PRISMA) [51]. The Prisma Checklist is available in the supplementary materials (S1 File), as well as the systematic review protocol (S2 File).

### Search strategy

PubMed, Embase and APA PsycInfo were searched, employing both controlled vocabulary and free text. Considering the debates surrounding the terminology associated with suicide, a wide range of words was selected to describe the spectrum of self-inflicted violence [52, 53]. All terms describing self-injury behaviour from ideation to self-harm and suicide have been included, with the exception of nonsuicidal self-injury (NSSI), as in a previous study [14]. Our goal was to identify articles that referred to interventions using the words intervention, implementation and program. We utilised a broad range of terms related to sex and gender, including sex and gender identity categories, social constructs related to gender, and words referring to sexual minorities. The decision was taken to incorporate them due to the established interconnection between sexual and gender minorities and gender studies in research, as well as in the databases keywords, in view of the link between sexism, heteronormativity and gender conformity. Other social positions and structures of oppression were examined in the selected articles, however these concepts were not included in the keywords. The final terms are available in the supplementary materials (S2 File).

Selected articles had to have been accepted for publication between 2000 and August 2022. Indeed, during this period, there was an increase in research on the relationship between gender and suicidal behaviour, following the seminal Gender Paradox study by Canetto and Sakinofsky [6, 54, 55]. We limited the search to studies in French and English. In addition, we included three previous systematic reviews on suicide prevention among AYA [14, 44, 45].

### Eligibility criteria

As in Robinson and colleagues review [14], studies were included if the majority of the sample consisted of AYA between the ages of 12 and 25 years. We chose this criterion in order to reflect the broader definitions of adolescence found in the literature, which include the beginning of adulthood [56]. The selected interventions were designed to prevent suicidal behaviour. These included crisis intervention for suicidal individuals, therapeutic programs, but also educational and awareness interventions, universal training, and e-health initiatives. A comparative design was not a requisite component of the study, and outcomes were not constrained. They could include changes in suicide and suicidal behaviour rates and trends, healthcare utilisation, or changes in knowledge concerning suicidal behaviour, among others.

Exclusion criteria included articles where NSSI was the main focus or outcome, as in Robinson and colleagues’ review who conceptualised it as a distinct behaviour [14]. Moreover it could have different gender implications [57]. We therefore decided to exclude these studies, along with those focusing on a risk factor for suicidal behaviour, and articles centred on mental health, anxiety or depression, in which suicidal behaviour was only a secondary outcome.

In addition, studies that employed exclusively screening techniques for suicidal risk or administered pharmacological treatments were excluded. Similarly, articles focusing on the evaluation of novel diagnostic tools and protocols were not included in the review. Interventions in adults involved in youth suicide prevention, including paediatric health professionals or caregivers, were also excluded.

The minimum criterion for a gendered approach was to identify a sex or gender identity category for the non-mixed samples, or to distinguish between a minimum of two sex or gender identity categories for mixed samples. This criterion was only applied during the second step of the review, during which the entire article was read.

### Selection process

After the search, duplicates were removed and the articles were screened and assessed for eligibility by two reviewers (JK and LL). Disagreements were resolved through discussion with a third reviewer (VB).

### Data collection process

To ensure a double extraction of gender data from all eligible articles, one reviewer extracted data from all articles (LL) while the other two shared the database (VB and JK). Disagreements were resolved through discussion of the three reviewers.

### Data items

The first data items collected were about the study, including information on participant type (student, patient, etc.) and their baseline characteristics (age range and mean age). Data on the intervention were gathered using the Gordon classification [58] which categorises preventive approaches as universal (for the entire population), selective (for a population with specific risk factors), or indicated (for people with an experience of suicidal behaviour), with the aim of determining whether there were differences in levels of gender integration depending on the intervention type. Similarly, and based on prior research on strategies to prevent suicidal behaviour [59–61], we categorised our studies according to the strategies they employed (e.g. school-based program, crisis intervention or general public information), in order to describe the main orientations of the programs. In addition, we extracted implementation environments (medical, school or community), as recommended in another review [14].

Each data was coded in accordance with the continuum proposed by Pederson and colleagues [1]. To determine the approach (gender-blind, sensitive, specific, or transformative) and the level of integration of gender-related content, we searched for gender items in each section of the scientific articles. In the introduction, we looked for prevalence and theoretical data, research questions, aims, and hypotheses from a gender perspective, as well as the construction and/or content of the intervention if specified. We conducted a comprehensive analysis of the sociodemographic data including their use in the text and the statistical models. Moreover, we investigated any reported differences in outcomes between sex/gender identity categories or other groups, and extracted any results reported with a gendered element. Lastly, we reviewed discussion and conclusion sections for gender-related content, responses to hypotheses, limitations, and opportunities for future research. Given our conceptualisation of gender through an intersectional lens, we searched for references to an intersectional framework, as well as sociodemographic data or contextual variables that could have been employed to analyse the interconnection of multiple social positions and structures. The result section reports on the categorisation of data based on this framework (see also Table 1). The list of items is available in the supplementary materials (S2 File).

**Table 1:** Coding for gender elements

	Blind	Sensitive (Minor)	Sensitive (Medium)	Specific	Transformative
Introduction^1^					
Prevalence and theoretical framework	No	Yes	Yes	Yes	Yes
Research questions	No	No	Yes	Yes	Yes
Study aims	No	No	Yes	Yes	Yes
Hypotheses	No	Yes	Yes	Yes	Yes
Development and content	No	No	No	Yes	Yes
Results					
Use of sociodemographic data^2^	Descriptive	Descriptive Analytical	Descriptive Analytical	Descriptive Analytical	Descriptive Analytical
Reported gender differences in outcomes^3^	No	Mentioned	Yes	Yes	Yes
Gender-related results^3^	No	Mentioned	Yes	Yes	Yes
Discussion					
Gender-related explanations^3^	No	No	Mentioned	Discussed	Discussed
Opportunities for future research^3^	Mentioned	Mentioned	Discussed	Discussed	Discussed
Limitations^3^	Mentioned	Mentioned	Discussed	Discussed	Discussed

^1^ Presence (Yes) or absence (No) of gender-related elements in each section

^2^ Descriptive: for simple description of the sample composition; Analytical: for data analyses or statistical modelling

^3^ Degree of mobilisation of gender-related elements in each section: No: complete absence, Mentioned: referenced but not further developed and discussed, Discussed: emphasised and developed

### Study risk of bias assessment

In conducting this review, we chose to systematically assess the integration of the concept of gender and the gender transformative health promotion framework, rather than focus on the statistical outcomes. Additionally, the sample was highly heterogenous and depicted a wide range of study designs, including qualitative, descriptive studies, quantitative randomised and non-randomised, with only a limited number of mixed methods studies. The diversity of designs made any quantitative comparison difficult.

The quality of the studies was assessed using the Mixed-Method Appraisal Tool (MMA-T), version 2018 [62]. This tool facilitated the measurement of the quality in a variety of study designs, including qualitative studies, randomised controlled trials, non-randomised controlled trials, descriptive studies and mixed methods.

### Data synthesis

This systematic review built on a framework synthesis [63, 64]. This method produces a type of systematic review that departs from the conventional approaches focused on effectiveness and quantitative comparisons. Instead, it adopts a qualitative approach, addressing theoretical enquiries and conceptual frameworks [65]. To ascertain the elements related to gender and their use, a narrative synthesis was performed regarding their integration of gender, following the continuum described by Pederson and al. [1]. The gender-blind articles confined the gendered approach to the description of the sample. The minor sensitivity to gender described articles wherein the gendered approach was problematised, yet the extant results and analyses were limited, while the articles with a medium sensitivity presented a few results and hypotheses concerning gender differences. To be categorised as gender sensitive, research should not only describe sample distribution and/or provide prevalence data in the introduction but also acknowledge gender differences [1] which we translated by using distribution data in sections beyond the sample description (such as their use in statistical models), or by investigating gender effects in data analysis. Gender sensitivity could also be achieved by problematizing gender as a specific issue. We therefore chose to classify as “minor sensitivity” articles conforming to a limited acknowledgement of gender and as “medium sensitivity” articles corresponding to the latter description. The gendered approach of the articles described as gender specific was focusing on specific categories, including young women, men, or LGBTI+ individuals. The last category was the one about gender transformative elements, emphasising interventions that not only focus on health outcomes but also actively challenge harmful gender norms. A comparative analysis of the main results related to gender is hereby presented. Additionally, the studies were also classified according to their setting, preventive approach and the prevention strategies developed, and compared regarding the gender classification.

## Results

### General results

**Fig. 1:**
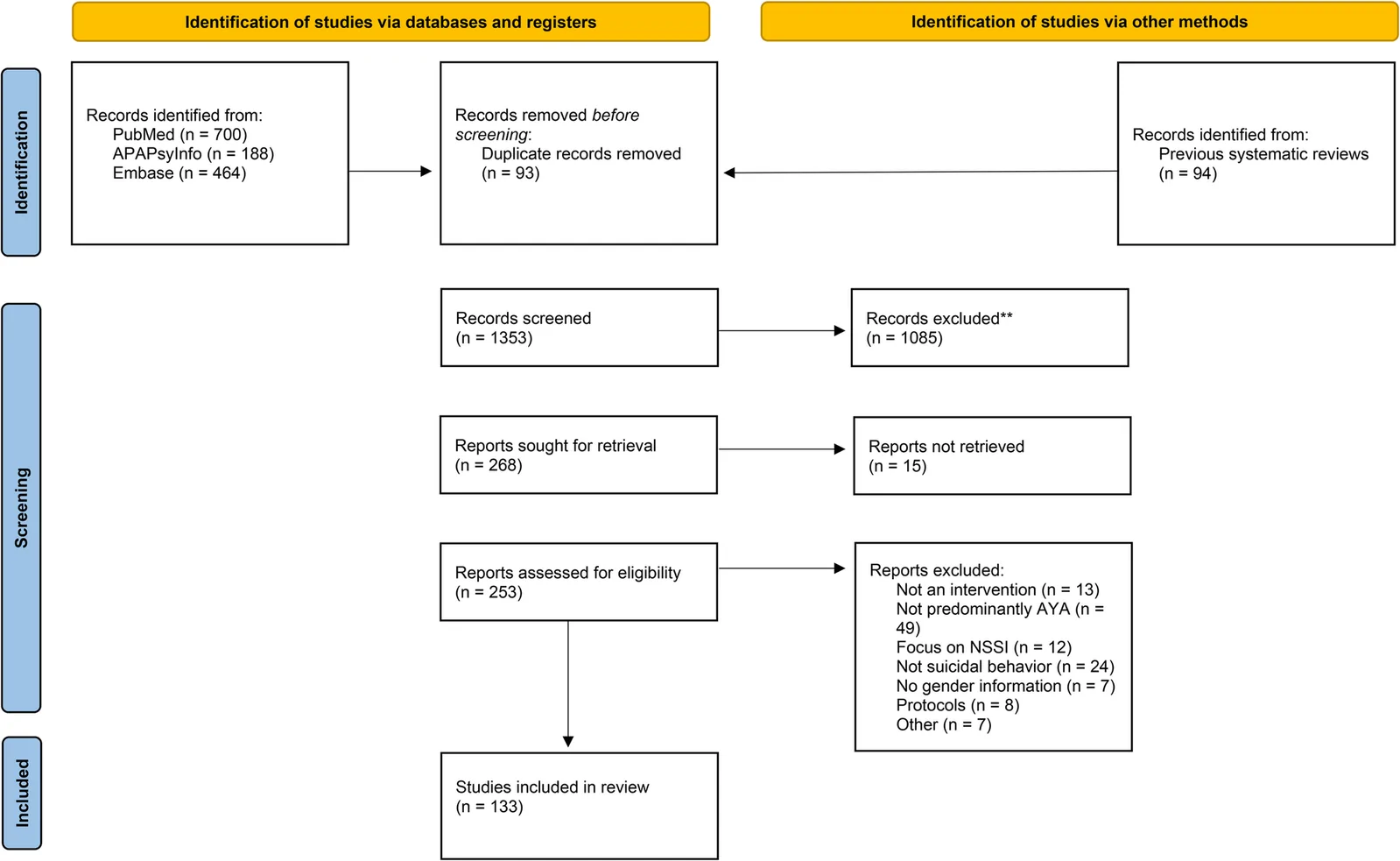
PRISMA Flow diagram of the inclusion process (template from Page et al., 2021)

From an initial number of 1,446 articles, 133 were included in the review (Fig. 1).

The majority of studies were conducted in medical settings, followed by schools and mixed settings (Table 2). The most developed preventive approach was “targeted” AYA with a history of suicidal behaviour, often treated during hospitalisation after suicidal behaviour or self-harm. The majority of the samples consisted of both young women and men.

**Table 2:** Synthesis of intervention characteristics (*n* = 133)

	Full sample (*n* = 133)	
	Number	Percentage
Setting		
Medical	65	49%
School	45	34%
Community	10	7%
Mixed*/other**	13	10%
Preventive approach		
Universal	34	25%
Selective	8	6%
Indicated	88	66%
Mixed Universal and Indicated	1	1%
Mixed Selective and Indicated	2	2%
Strategy***		
General public information	9	7%
Media, telephone and internet services	32	24%
Awareness and education	47	35%
Gatekeeper training	9	7%
School-based program	32	24%
Management/Treatment	93	70%
Safety planning	9	7%
Crisis intervention	6	5%
Care coordination	20	15%
Contact-information/follow-up care	12	9%
Screening	17	13%
Peer-to-peer support	18	14%

^1^Mixed: more than one setting/recruitment strategy

^2^Other: workplace, military or institutional places

^3^Combination possible, hence total may be more than 100%

“Management and Treatment” was the most frequently coded prevention strategy (70% of the articles), followed by “Awareness and Education” (35%), “School-based program” (24%) and “Media, Telephone and Internet Services” (24%). “Awareness and Education” and “School-based program” strategies were often combined. These results are consistent with the predominance of a biomedical lens in the production of suicide prevention approaches [47]. The level of gender integration was similar for all preventive approaches or strategies.

The tables presenting the studies can be found in the supplementary materials (S3-S7 Files), as is the list of included references (S8 File).

### Gender focus

**Fig. 2:**
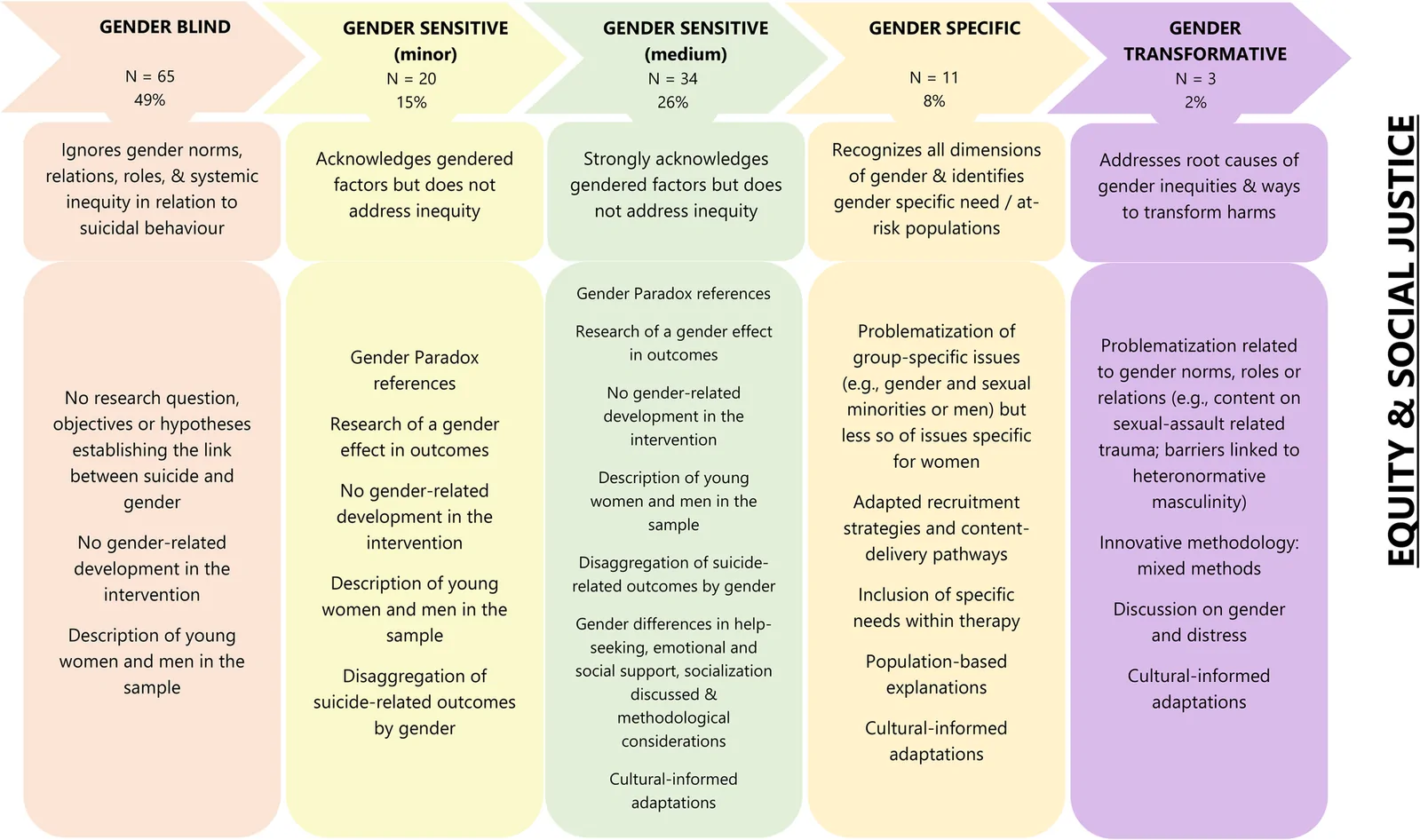
Gender transformative continuum for suicidal behaviours in adolescents and young adults (adapted from Greaves et al. [66])

Most articles only utilised gender-related information to describe the sample. 70% of the articles used the word gender, 41% the word sex, and 22% used both, most often interchangeably. 96% of the articles primarily referred to the categories of female and male. 10% of articles reported the youth sexual orientation, 14% used words referring to transgender individuals and nonbinary categories (genderqueer; nonbinary people; other), 1% of articles referred to intersex people. The terms sex and gender were used interchangeably. There were few terms referring to gender-related dimensions or social constructs (roles, norms, sexism, etc.). The term ‘gender’ often referred to the gender identity dimension without details on how the data was collected (e.g. self-declaration, medical records, etc.). Moreover, the sex/gender identity was predominantly reported in a binary manner with the exception of 15 articles (11%) which considered the use of non-binary categories. The articles are examined in accordance with the gender transformative continuum [1], we made an adaptation of the continuum related to our topic (Fig. 2).

### Heterogeneity in designs and quality in the continuum

According to the continuum measuring gender integration, gender-blind articles were predominantly quantitative designs, consisting mainly of randomised and non-randomised trials. The remaining categories, namely gender sensitive, gender specific and gender transformative, displayed a broader variety of designs.

The quality assessment process was similarly complex. Using the Mixed-Method Appraisal Tool (MMA-T), most articles received a score of 2 or 3 on 5-point scale, with no notable difference observed across the continuum. The results are available in the Supplementary Information (Appendix E, Tables EI – E5).

### A majority of gender-blind articles (*n* = 65, 49%)

The majority of the sample was gender-blind (49%): gender was assessed as a binary variable through the sex or gender identity categories, the demographics were described but not used further. Moreover, the articles highlighted a limitation of their sample which was predominantly composed of women (23%) and lacked diversity (12%). However, in our sample, gender approaches gained greater importance from 2016 onwards.

### Continuum of gender sensitivity (*n* = 54, 41%)

#### Minor sensitivity to gender (*n* = 20, 15%)

This set of articles used sex or gender identity categories as a variable in the statistical models, either as an adjustment variable or in search of a moderator effect [67–80]. The available data depicted the use of the sex/gender identity categories as a binary categorical variable, the details about their use in the statistical models were limited due to the standardised format for scientific medical literature. However, these articles did not rely on theoretical content or provide a thorough explanation for the search for gender effects. One article referred to prevalence data in the introduction to justify the need for intervention regarding the rise in suicidal behaviour for adolescents in general [75] and two cited previous research articles that identified sex/gender as a significant factor with no further theoretical argumentation [74, 81]. As such, gender was not a central concept, but rather one that authors may feel strongly encouraged to use as a necessary step in their research, yet one that they mention without analysing or discussing it further.

Articles mentioning gender in results and discussion sections highlighted slight differences, mostly in an outcome and without explanation [73, 82, 83]. For the most part, there were no results to discuss regarding gender since no significant statistical effect was highlighted when searching for moderation or differences [68, 71, 78, 80, 84]. Adrian et al. indicated that the lack of analysis or further exploration in their study was due to the limited enrolment of young men which did not allow for moderator effect analysis of sex [81]. Similarly other sociodemographic data, such as race/ethnicity, were reported and added to the statistical models. No theoretical rationale was provided for their inclusion with the exception of Han et al. vis-à-vis cultural adaptation for implementation in both Australia and China [74].

#### Medium sensitivity (*n* = 34, 26%)

In this category, gender issues were discussed from the introduction with sex/gender identified as an important potential moderator in seven studies [85–91]. Four studies focused in part on the comparison between women and men [92–95] and three studies formulated hypotheses about greater effectiveness or engagement in the intervention for young women linked to differences in help-seeking behaviour or suicidal behaviour [86, 87, 91]. In this context, differences between young women and men in program outcomes were expected, both at baseline and after the program. However, five articles that analysed a large sample of data explicitly underlined an absence of significant interactions/effects [87, 91, 96–98].

Several studies reported some (few, in general) differential effects of interventions depending on gender. For example, the results reported by King et al. showed that young men perceived receiving mental health treatment as being less stigmatised after the intervention but this was the only measure with a significant gender difference [88]. Two other studies also concluded that young men were more responsive to treatment [95], particularly when identified as high suicide risks [93]. However, it should be noted that in at least one study, the proportion of men was very small (7/49) [95]. Finally, seven articles found more benefits and greater effectiveness for young women after the program [86, 94, 99–103].

We noted that the cause for gender differences or variations in outcomes was not really discussed or captured. Eleven articles provided methodological explanations for the observed differences or lack thereof, such as a small sample size or a predominance of young women [90, 95–98, 103–108]. According to Haas et al., the higher participation of young women in the programs could be attributed to recruitment criteria such as depressive symptoms which are identified in the medical literature as being more prevalent in women [96].

Moreover, results that show no statistical gender difference [87, 91, 96–98] may be explained by the influence of other variables, including age, race or class, which have also been demonstrated to be associated with distress [11, 33]. The context and implementation of the intervention, such as the setting and the interactions between participants and animators, may also play a significant role in the outcomes observed. It is worth noting that in the absence of detailed descriptions of the interventions in the papers, it is difficult to analyse further those aspects.

Furthermore, in the discussion sections, theoretical contribution was focused on differences in socialisation and health-related behaviour. In the Thompson et al. study on group-based anger management, the difficulty to challenge the stereotypic behaviour of young men in public was identified as a limit [92]. King et al. elaborated on the differences in getting emotional and social support [86]. Flynn et al. referred to social psychology, specifically gender role theory [109] to explain the lack of help-seeking in young men in their sample [101]. According to this approach, gender is integrated by individuals from childhood and femininities and masculinities imply ways of behaving according to the sex assigned at birth [109], for instance, regarding help-seeking behaviour which are less encouraged in boys [54]. However, these theoretical elements remain limited and were not integrated into the programs and their evaluations. This emphasis on differences focused exclusively on young women vs. men in the results section highlights a restricted interpretation of gender, which consequently limits its utilisation in the articles. This phenomenon may also be explained by the absence of theoretical problematisation in the introduction.

In addition to gender, it should be noted that the collected articles included other sociodemographic data such as social background, race/ethnicity, and parental information, as well as contextual characteristics such as school or community features. Several articles used this data to describe the sample and the differences between groups based on race/ethnicity, but none explicitly developed an intersectional approach [36]. There were, however, a few leads through culturally-informed interventions. For instance, Allen et al. incorporated traditional knowledge from Alaska natives into their program aimed at preventing suicide and alcohol consumption [110]. Three additional articles also examined cultural adaptations [105, 111, 112] and two articles suggested the importance of taking race/ethnicity into account regarding the outcomes in their samples [90, 107].

#### Gender specific intervention (*n* = 11, 8%)

Eleven articles (for ten distinct interventions) developed a gender specific approach that focused on an identified at-risk population. Four of them focused on young men, four on young women and three on young LGBTI+ people. These articles mobilised more theoretical content to justify tailored interventions and related adaptations in the programs.

Interventions focused on young men have typically taken place in non-mixed or predominantly masculine environments such as a construction workplace [113], a military context [114], a football club [115], with the exception of a school-based intervention [116]. The justification for a specific intervention was based on the observed differences between young women and men in mental illnesses, mental health treatment and suicidal behaviour. Specifically, Broadbent et al. introduced their study with data on the rise of suicide in young men [113], while Calear et al. started with an emphasis on the lack of help-seeking behaviour in this population owing to the stigmatisation surrounding mental health of boys and men and masculine norms [116]. These authors also developed an explanation from social psychology on gender norms and the pressure to conform and their effect on emotion management and help-seeking [116]. Interventions have been adapted to include changes in content delivery pathways [113–116], the use of expected masculine role models such as athletes [116] and challenging stereotypes during group interventions [115, 116]. One article also focused on indigenous men and included cultural adaptations [115].

In our sample, interventions targeting LGBTI+ youth were based on data describing them as an at-risk population owing to higher rates of suicidal behaviour and specific risk factors such as stigmatisation and discrimination as well as barriers to care [117–119]. Two articles developed a targeted approach to prevention and adapted therapy content to include the needs of LGBTI+ patients [117, 119]. This involved changes in therapy structure to allow for more time with parents, integration of family reactions to sexual orientation, and strengthening family connections [117, 119]. One recent intervention adapted the recruitment efforts to target the LGBTI+ population through social media [118].

As for interventions specific to women, interestingly, Gueddes et al. as well as Rotheram-Borus et al. did not problematise the fact that their samples only included women [120, 121]. The remaining two articles by Humensky et al. described a program which combined medical and community settings to target Latina adolescents receiving mental health treatment [122, 123]. This program addressed specific risk factors, such as family conflicts, societal expectations, and gender roles [122, 123]. This approach was considered promising to help with parental conflicts that lead to distress. Rotheram-Borus et al. [120], as well as the two articles by Humensky et al. [122, 123], focused on Latina women. However, only the latter two developed a comprehensive approach in their program [122, 123].

Thus, we may notice that for LGBTQI+ individuals and young men, gender was problematised as a contributing factor to distress and can thus be considered as an object of intervention. However, “gender” is understood in a sense that targets certain specific populations, potentially to the detriment of others (e.g., young women).

#### Gender transformative elements (*n* = 3, 2%)

None of the articles in the sample explicitly developed a gender transformative approach as defined by Pederson et al. However, we identified elements that acknowledged inequalities and attempted to challenge them. Three articles published since 2016 have provided (with more or less detail) a description of the integration of gender and the adaptations made in the interventions to address gender-related issues.

In their Intensive Outpatient Program (IOP), Salk et al. introduced group content about substance use and trauma following sexual assault [124]. This was justified by the concerning prevalence data regarding women who have been sexually assaulted while in college [124]. The study showed promise in the care of suicidal college students and suggested developing content about discrimination, particularly focused on sexual and gender minorities [124]. However, the article did not provide much elaboration on the program or its evaluation.

Duarté-Vélez et al. developed a program based on cognitive behavioural therapy (CBT) adapted to suicidal behaviour [125]. The program was constructed with a feminist approach, aiming to challenge gender roles by acknowledging stereotypical parenting expectations of young girls and boys during adolescence [125]. Although the program showed promise in improving retention and reducing suicide ideation among participants, Duarte-Vélez et al. insisted on the small sample size (11 individuals) as a limitation [125] and argued for further exploration to determine its overall effectiveness [125]. Their study is the only one in this category developing cultural adaptations by emphasising gender roles, norms, and minority acceptance related to Latino culture [125].

Finally, Honea et al. developed a program through a theatre workshop aimed at challenging stigma around mental health and gender as well as help-seeking barriers, particularly among men [126]. Young adults participated in a workshop organised to write the narrative based on their personal experiences of suicidal behaviour and to share prevention messages during a performance and an exchange with the public [126]. The program was evaluated through a qualitative study with a gender lens [126]. The researchers identified the need to address heteronormative masculinity to encourage help-seeking in young men [126]. Although the authors acknowledged that the sample may not be representative and the participants may be considered atypical, the findings were interesting in reflecting on gender norms in suicide prevention strategies [126]. The evaluation’s qualitative design facilitated an analysis of participant internalised gender stereotypes and norms related to suicidal behaviour and attitudes towards a distressed peer as well as the need to challenge them [126].

Transformative approaches within the fields of health and implementation science have only recently begun to emerge, which may partly explain the limited number of studies identified. Moreover, the development of these approaches may depend on methodological frameworks that were less frequently represented in the studies analysed.

## Discussion

The objective of the present study was to conduct an analysis of the current use of gender in the youth suicidal behaviour prevention programs and to describe gender transformative elements when present. It should be borne in mind that the review did not assess the outcomes of the interventions; therefore, no conclusion can be drawn regarding their efficacy. However, we believe this study is an important first step to question the utilisation of gender and of the gender transformative framework in the current research practices.

Based on the Pederson et al. framework [1], our results show a limited gendered approach in suicide prevention since nearly half of our sample was gender-blind (46%). The incorporation of a gender perspective largely depended on how the authors employed the term gender and varied considerably across studies. Although some studies included non-binary data, the methods of data collection and their subsequent use in statistical analyses present several challenges. Recent studies have described the most effective methods for operationalising gender identity in quantitative research [127–129]. The use of a third category (often referred to as ‘non-binary’ or ‘trans’) could have limitations due to its non-exclusive nature in comparison with the categories of women and men [128]. The advantages and disadvantages of using multiple categories, free text, or the two-question method to assess gender identity and sex assigned at birth, or trans experiences, should be considered when deciding on an approach [127, 128]. Consequently, articles that were gender-blind confined gender to descriptive data.

The articles that were not gender-blind were primarily distributed based on varying gender sensitivities (38%). The prevailing approach was based on the differences between young women and men. They typically referred to gender differences in the prevalence of suicidal behaviour and risk factors and analysed disaggregated efficacy data to identify potential gender effects. Although some articles reported differences in outcomes between young women and young men, it is challenging to explain these variations without considering other individual and structural characteristics or contextual factors. Therefore, there was no inclusion or integration of data related to constructs such as gender norms, roles, relations or institutions in connection with suicidal behaviour into the gender sensitive prevention programs themselves. Consistent with the findings of a narrative review [39] and two systematic reviews [14, 45], a considerable proportion of the studies used samples consisting mainly of women. Similarly, among the limited differential effects observed, young women exhibited a greater benefit than men. The explanations provided for these partial results were still limited and focused particularly on the methodology used or the hypothesis related to socialisation, specifically in terms of seeking help and accessing care differentially [54]. However, this hypothesis remained untested in our sample and requires further analysis. Two major limitations were often listed to justify the absence of gender analysis in the study sample: small sample sizes and over-representation of young women. These limitations made it difficult to conduct statistical comparisons, especially since the analyses were mainly quantitative. Consequently, the discussion of gender elements was constrained to a binary perspective that accentuated differences, thereby limiting a gendered approach to the comparison of women and men. A number of important interrogations have been raised in the gender sensitive articles that warrant consideration in future studies, such as questioning the hypothesis of a greater effectiveness and engagement of young women in programs, as well as differences in help-seeking behaviours and social support. However, the current approach, which involves the investigation of effects moderated by sex/gender identity, appears to be limited, with only a few results reported. Furthermore, a gendered approach cannot be limited to examining potential differences in efficacy between young women and men, as this tends to overlook the influence of social structures on the production of disparities.

Additionally, gender specific articles devoted a larger portion of their content to gender issues (8%). The main purpose of this problematisation was to provide a rationale for prioritising vulnerable populations. This resulted in the creation of targeted recruitment strategies and the recognition of harmful gender stereotypes and discrimination in program development, particularly for LGBTI+ youth and young men. Nonetheless, the focus on at-risk populations may inadvertently contribute to the perpetuation of stereotypes and gender norms [130]. In particular, to encourage the participation of young men in the samples, specific recruitment methods and interventions were developed. Several ideas were drawn from masculine norms and associated figures. However, Calear et al. explained that the utilisation of traditional masculine values as a basis for recruitment may have limited the participation of young men who do not align with these values [116]. Indeed, suicidality could be associated with the adherence to hegemonic masculinity ideals, such as emotional suppression [6, 131]. This has influenced the development of prevention campaigns targeting men by encouraging them to “talk”, which is often viewed as a major barrier to seeking help [132]. Nevertheless, Chandler has shown the importance of analysing the context in which speech is initiated and its effects on individual status (within peer groups, families, etc.), the interest in defining what is considered unspeakable, and in considering social inequalities and gender in prevention and prevention politics [132, 133]. The literature has also demonstrated the significance of the LGBTI+ suicidal behaviour framing and the necessity of addressing determinants that extend beyond an individualistic approach [134, 135]. The identification of gender norms and social structures related to suicide and help-seeking care should make it possible to challenge them.

Finally, while many articles in the sample collected socio-demographic data and discussed their use, the approach that combines gender with other social positions, such as race/ethnicity or class and with systems and structures of power [36, 136], was not well-developed. The use of such an intersectional framework could be fruitful and essential in elaborating prevention programs and analysing their effects [137, 138], as recommended in the gender transformative framework [1] or by authors pleading for more integration of sociological approaches in public health research [139, 140], and encouraged in recent works on LGBTI+ suicide prevention [35]. This approach has the potential to facilitate the understanding of the complex intricacies between multiple identities and suicidal behaviour, while also actively contribute to the advances on social justice [36]. The absence of an intersectional understanding of gender may be explained by limited awareness of both the framework and the literature on its operationalisation within public health. The relative novelty of the intersectional approach, as highlighted in the works of Lisa Bowleg and the establishment of a dedicated section in the *American Journal of Public Health* in 2021 may also provide an explanation [36, 141].

Our review has some limitations. First, focusing solely on suicidal behaviour may have caused us to overlook innovative articles in broader mental health research from a gender perspective. Moreover, our analysis was limited to English and French literature which may have also restricted our ability to fully appreciate works from other countries and contexts. The present study included only studies published in peer-reviewed journals and indexed in the three selected databases. However, prevention interventions published in peer-reviewed journals constitute only a subset of the available interventions. Other sources, such as practitioner journals and research or work reports, were not included in this study. A final point to emphasise is that this work was completed in 2022. Recent studies have since been published and could not be included into the analysis [142]. Overall, these studies do not appear to fundamentally challenge the conclusions of our research. Nevertheless, some of them offer a more in-depth gender perspective, particularly in relation to sexual and gender minorities [143, 144], or to explicitly adopt a gender transformative approach targeting young men [145]. They should certainly be taken into account in future analyses.

Despite these limitations, this is the first systematic review to appraise gender in suicide prevention using the gender transformative framework. Consequently, it is based on a large sample of studies which has enabled us to identify important leads for future research that we describe below. Some of these leads also rely on ideas developed by the few papers included in the present review that exhibited some gender-transformative elements. A summary table of the recommendations for research and practice is available at the end of the article (Table 3).

**Table 3:** Integrating gender-transformative approach in suicide prevention interventions: recommendations for research and practice

Issues		Recommendations
Theoretical issues	Limited theoretical engagement with gender	- Strengthen the theoretical engagement with gender by conceptualising it as an analytical category - Consider drawing on frameworks such as: o Gender transformative health promotion [1] o Critical suicide studies [48, 50, 146]
	Limited use of gender constructs in relation to suicidal behaviour	- Consider drawing on an interdisciplinary body of literature and mobilising concepts such as: o Individual, contextual and structural factors when explaining distress and suicide [35, 139] o Gender norms and roles, interpersonal and intergroup relations, institutionalised gender, considered within an intersectional framework [22] o Structural inequalities (e.g. racism, sexism, heteronormativity) [147] - Acknowledge the range of theoretical perspectives on suicidal behaviour and corresponding terminology
Intervention development issues	Insufficient attention to gender-related aspects in intervention content, dissemination methods, and professionals’ positioning	- Explore gendered risks (e.g. sexual violence, adherence to harmful gender norms) and protective factors (e.g. social support) influencing suicidal behaviour - Consider mobilising gender norms and roles, interpersonal and intergroup relations, institutionalised gender in the intervention and its implementation (e.g. risk of perpetuating gender stereotypes in interventions focusing on young men)
	Predominance of approaches targeting only individuals who have previously engaged in suicidal behavior	- Need for the development of universal and selective approaches [58] - Expansion of intervention settings (e.g. school, community, etc.) [148]
	Essentialisation of gender differences	Critically question gender norms and roles, interpersonal and intergroup relations, institutionalised gender, considered within an intersectional framework in intervention content and design
Evaluation and analysis issues	Methods of gender data collection and operationalisation	Define measures based on literature, including the use of multiple gender identity categories [127–129] and gendered variables to capture broader gender-related dimensions [149]
	Limited consideration of sampling issues	Critically examine the relationship between gender and sampling (e.g. due to gendered socialisation)
	Hypothesis of a greater effectiveness and engagement for young women compared to young men without further investigation	Analyse and evaluate the intervention beyond statistical comparisons between young women and men
	Constraints of quantitative measures and moderation analyses based on sex or gender identity	Draw on mixed-methods and interdisciplinary approaches, as well as the literature on complex interventions, to provide a more comprehensive evaluation of intervention [38, 150]
	Consideration of gender as the sole analytical dimension	- Integrate an intersectional perspective in the analyses [36, 136, 141] - Critically reflect on the impact of social inequalities, taking into account individuals’ unequal positioning within social structures (e.g. racism, sexism, heteronormativity, and broader political contexts)

This review is the first one to examine how gender is addressed in suicide prevention. In other areas of public health, gender transformative approaches have been limited, particularly in relation to the prevention of the use of psychoactive substances [151]. Sexual health promotion has historically been more attentive to gender dimensions, yet important challenges remain, such as the need for stronger theoretical engagement [152]. The main criticisms and challenges reported in these interventions, including an overemphasis on individual-level change, a lack of rigorous methods to assess gender-related outcomes, and ongoing difficulties in documenting implementation processes and demonstrating evidence of transformation, echo the findings of our own research [152, 153].

The findings of this review have prompted to the formulation of new hypotheses and questions, which may inform future research on the integration of gender studies in suicide prevention. Indeed, examining gender within suicide prevention interventions calls for a paradigm shift in both our research methodologies and the way we frame our research questions. A first step could be to broaden our theorical perspectives by drawing on insights from other disciplines and fields, and by critically re-examining how suicidal behaviour itself is conceptualised.

From a theoretical perspective, we suggest that the integration of the principles of a gender transformative approach resonate deeply with the work of critical suicide studies, an analytical framework that was not mobilised in the sample either. Suicide has been conceptualised through different models and disciplines and critical suicide studies have prompted the adoption of new frameworks as in a social justice analysis [48]. Suicide prevention through a gender transformative approach could benefit from this field, particularly in light of the necessity to integrate the effects of social inequalities. This implies acknowledging the unequal positioning of individuals in social structures, such as racism, sexism, and heteronormativity, as well as political contexts that produce violence [146, 154], and by considering such concepts as norms and beliefs/ideologies, social roles, and the role of relationship dynamics, social processes and institutions in maintaining or challenging inequalities, as previously acknowledged in tobacco prevention [22, 130]. Critical suicide studies have advocated moving beyond the prevailing biomedical and individualistic model of suicide research and prevention [49, 146]. This shift involves the recognition of different theoretical frames and the refinement of associated terminology, thereby redefining research on suicide [49, 50, 146, 147, 155].

Secondly, the content of the programs, method of transmission and positioning of professionals throughout prevention should be an area of interest. To this end, exploring gendered risk and protective factors could be valuable. For example, in the review, Salk et al. advocated for the creation of content that addresses the issue of sexual violence [124]. Indeed, trauma has been identified as a risk factor for suicidal behaviour in adolescents [156], especially in cases of sexual violence [157]. The trauma-informed approach sensitises professionals to “cultural, historical and gender issues” as a fundamental principle [158] and has been associated with a gendered and intersectional approach for the development of equity [159, 160].

Similarly, in the review, Duarté-Vélez et al. developed an intervention that aligns with a feminist orientation, with the objective of challenging prevailing stereotypes and norms within the context of the lives of the young patients and therapy [125]. This idea could be linked to critical suicide studies and the creation of a new therapeutic environment [161] as in narrative therapy [49]. This challenges the exclusive focus of healthcare professionals on risk management and individual or medical issues. The advent of novel approaches to care presents an opportunity to establish new spaces of prevention and professional positions.

Moreover, the expansion of intervention settings to include schools and communities has the potential to facilitate the advancement of gender approaches by integrating social determinants [162]. Although universal and selective approaches have been relatively under-researched in suicide prevention, as evidenced in our sample, they have recently been encouraged and would imply considering the determinants of health and social structures in public health policy [35, 163].

Finally, the evaluation of the programs could benefit from innovative methods and the introduction of approaches from other disciplines, as well as other fields and concepts, that may facilitate the integration of a gendered perspective. The apparent absence of effect in some studies regarding gender may stem from methodological limitations rather than from the absence of gender related elements. While the evidence-based paradigm continues to dominate public health research, emerging scholarship highlights the value of integrating complementary approaches and mixed outcome measures to more accurately assess complex interventions [164, 165]. For instance, gender could be measured not solely on the basis of gender identity but also in relation to gender constructs such as roles, norms and relations [22], and their cultural contextualisation [6, 55]. This may be achieved through the use of tools developed in the context of health and public health studies [149, 166]. This approach has the potential to facilitate the analysis of gender beyond the conventional binary distinction, encompassing gender-related variables not limited to the sex/gender identity one. Furthermore, this methodology could allow for analysis even in samples with no gender balance.

Fisher et al. have also called for the development of evaluation models that integrate the concept of complexity, measuring the influence of social gender norms in addition to the impact of interventions on health outcomes [38]. Their review of programs using a transformative approach highlighted the beneficial effects of interventions that also target gender socialisation, particularly when gender-based violence is concerned [38]. The concept of complexity allows for the consideration of multifactorial aetiology in health problems and the gathering of information about implementation of the intervention, contextual factors that facilitate or hinder use, as well as the mechanisms of action and processes involved in the targeted change [167]. These dimensions are related to gender and distress because they help to understand why certain populations are less likely to participate in suicide prevention interventions, the context that produces distress and behaviour change, and the mechanisms of action themselves which can be influenced by gender.

Furthermore, gender transformative school-based sexuality education integrating a complexity framework employed qualitative evaluation methods [168] considered more suitable for generating measures of complexity than randomised controlled trial alone [167]. Indeed, in the review, Honea et al. integrated a mixed methods evaluation of their program and participative methods, allowing to get insights into participants. Critical suicide studies also suggest that suicide prevention requires the development of mixed methods that combine quantitative analyses with qualitative investigations and interdisciplinary collaborations [146]. This approach would facilitate a more comprehensive understanding of suicidal behaviour and its prevention from a gender perspective. Last, as with other areas of intervention, co-construction and participatory research approaches appear to be particularly beneficial for discussing gender norms [154], as emphasised by one article in the sample [126]. White recommends that suicide prevention interventions in schools should not only focus on detecting signs of distress, but also on exploration and exchange involving young people as experts by drawing on written sources or testimonials [154]. Critical suicide studies have highlighted the need to incorporate novel methodologies and the experiences/perspectives of those who have been directly affected [48, 138]. While this process is often impeded in this area, the most recent guidelines represent promising opportunities [169].

In conclusion, the findings of the review indicate that the application of gender-based approaches in current suicide prevention strategies for adolescents and young adults remains limited. However, it also identified potential avenues for future programs. As evidenced by numerous studies and emphasised by various institutions, there are potential inequalities and variations in suicidal behaviours according to various social positions and structures. The integration of gender and intersectionality frameworks into programming could prove valuable for developing new approaches to preventing suicidal behaviour in adolescents and young adults. Thereby, this approach could benefit from various literature sets, including the gender transformative framework and the field of critical suicide studies.

## Supplementary Information

Supplementary Material 1: Appendix A: Prisma 2020 – Checklist. Appendix B: Systematic review protocol. Table B1. List of keywords used in the research. Table B2. List of data items. Appendix C: Details on each studies by gender category. Table C1: Gender-blind studies (*n* = 65). Table C2: Studies with minor sensitivity to gender (*n* = 20). Table C3: Studies with medium sensitivity to gender (*n* = 34). Table C4: Studies with a gender specific approach (*n* = 11). Table C5: Studies with gender transformative elements (*n* = 3). Appendix D: References of included studies (*n* = 133). Appendix E: Quality appraisal. Table E1: Qualitative studies (*n* = 5). Table E2: Quantitative randomised controlled trials (*n* = 56). Table E3: Quantitative non-randomised studies (*n* = 65). Table E4: Quantitative descriptive studies (*n* = 1). Table E5: Mixed methods studies (*n* = 6).

## Data Availability

The study is a systematic review of the literature, no primary data were included. The references of all included studies are available in the Supplementary Information.

## Footnotes

1 Throughout the text, we have retained the terminology used by the original authors of the papers reviewed, which most often refers either to sex rather than gender identity, or to gender to denote the sex category assigned at birth.
