# Supplementary material for: Gender in suicide prevention in adolescents and young adults: a systematic review
Source: BMC Public Health. 2026 Jul 9;26:2748. doi: 10.1186/s12889-026-28223-1 (PMC13632111; doi:10.1186/s12889-026-28223-1)
Supplement: Supplementary file 1 [file 12889_2026_28223_MOESM1_ESM.docx]

**Gender in suicide prevention in adolescents and young adults: a systematic review**

**Supplementary material**

**Authors: Léa Loubet^a^, Virginie Bonnot^b^, Joëlle Kivits^a^**

^a^	Université Paris Cité, Inserm, ECEVE, F-75010 Paris, France

^b^ Université Paris Cité, Laboratoire de Psychologie Sociale, F-92100 Boulogne-Billancourt, France

*Email address: lea.loubet@inserm.fr*

Table of content

[**Supplementary material A: Prisma 2020 – Checklist**	2](#_Toc228543305)

[**Supplementary material B: Systematic review protocol**	8](#_Toc228543306)

[Table B1. List of keywords used in the research	8](#_Toc228543307)

[Table B2. List of data items	10](#_Toc228543308)

[**Supplementary material C: Quality appraisal**	12](#_Toc228543309)

[Table C1: Qualitative studies (n = 5)	13](#_Toc228543310)

[Table C2: Quantitative randomised controlled trials (n = 56)	14](#_Toc228543311)

[Table C3: Quantitative non-randomised studies (n = 65)	19](#_Toc228543312)

[Table C4: Quantitative descriptive studies (n = 1)	25](#_Toc228543313)

[Table C5: Mixed methods studies (n = 6)	26](#_Toc228543314)

[**Supplementary material D: Details on each studies by gender category**	27](#_Toc228543315)

[Table D1: Gender-blind studies (n = 65)	27](#_Toc228543316)

[Table D2: Studies with minor sensitivity to gender (n = 20)	47](#_Toc228543317)

[Table D3: Studies with medium sensitivity to gender (n = 34)	54](#_Toc228543318)

[Table D4: Studies with a gender specific approach (n = 11)	66](#_Toc228543319)

[Table D5: Studies with gender transformative elements (n = 3)	71](#_Toc228543320)

[**Supplementary material E: References of included studies (n = 133)**	74](#_Toc228543321)

# **Supplementary material A: Prisma 2020 – Checklist**

*The PRISMA 2020 Checklist comprises 27 items representing each section of a systematic review report, in accordance with the PRISMA 2020 Statement (Page 2021)*

| **Section and Topic** | **Item #** | **Checklist item** | **Location where item is reported** |
| --- | --- | --- | --- |
| **TITLE** | | |  |
| Title | 1 | Identify the report as a systematic review. | Title page |
| **ABSTRACT** | | |  |
| Abstract | 2 | See the PRISMA 2020 for Abstracts checklist. | Abstract |
| **INTRODUCTION** | | |  |
| Rationale | 3 | Describe the rationale for the review in the context of existing knowledge. | Introduction  Para 1 - 5 |
| Objectives | 4 | Provide an explicit statement of the objective(s) or question(s) the review addresses. | Introduction  Para 6 |
| **METHODS** | | |  |
| Eligibility criteria | 5 | Specify the inclusion and exclusion criteria for the review and how studies were grouped for the syntheses. | Methods  Eligibility criteria |
| Information sources | 6 | Specify all databases, registers, websites, organisations, reference lists and other sources searched or consulted to identify studies. Specify the date when each source was last searched or consulted. | Methods  Search strategy |
| Search strategy | 7 | Present the full search strategies for all databases, registers and websites, including any filters and limits used. | Methods  Search strategy |
| Selection process | 8 | Specify the methods used to decide whether a study met the inclusion criteria of the review, including how many reviewers screened each record and each report retrieved, whether they worked independently, and if applicable, details of automation tools used in the process. | Methods  Selection process |
| Data collection process | 9 | Specify the methods used to collect data from reports, including how many reviewers collected data from each report, whether they worked independently, any processes for obtaining or confirming data from study investigators, and if applicable, details of automation tools used in the process. | Methods  Data Collection process |
| Data items | 10a | List and define all outcomes for which data were sought. Specify whether all results that were compatible with each outcome domain in each study were sought (e.g. for all measures, time points, analyses), and if not, the methods used to decide which results to collect. | Methods  Data Items  Supplementary material B, Table B2 |
|  | 10b | List and define all other variables for which data were sought (e.g. participant and intervention characteristics, funding sources). Describe any assumptions made about any missing or unclear information. | Methods  Data Items  Supplementary material B, Table B2 |
| Study risk of bias assessment | 11 | Specify the methods used to assess risk of bias in the included studies, including details of the tool(s) used, how many reviewers assessed each study and whether they worked independently, and if applicable, details of automation tools used in the process. | Methods  Study risk of bias assessment (NR) |
| Effect measures | 12 | Specify for each outcome the effect measure(s) (e.g. risk ratio, mean difference) used in the synthesis or presentation of results. | NR |
| Synthesis methods | 13a | Describe the processes used to decide which studies were eligible for each synthesis (e.g. tabulating the study intervention characteristics and comparing against the planned groups for each synthesis (item #5)). | NR |
|  | 13b | Describe any methods required to prepare the data for presentation or synthesis, such as handling of missing summary statistics, or data conversions. | NR |
|  | 13c | Describe any methods used to tabulate or visually display results of individual studies and syntheses. | NR |
|  | 13d | Describe any methods used to synthesize results and provide a rationale for the choice(s). If meta-analysis was performed, describe the model(s), method(s) to identify the presence and extent of statistical heterogeneity, and software package(s) used. | NR |
|  | 13e | Describe any methods used to explore possible causes of heterogeneity among study results (e.g. subgroup analysis, meta-regression). | NR |
|  | 13f | Describe any sensitivity analyses conducted to assess robustness of the synthesized results. | NR |
| Reporting bias assessment | 14 | Describe any methods used to assess risk of bias due to missing results in a synthesis (arising from reporting biases). | NR |
| Certainty assessment | 15 | Describe any methods used to assess certainty (or confidence) in the body of evidence for an outcome. | NR |
| **RESULTS** | | |  |
| Study selection | 16a | Describe the results of the search and selection process, from the number of records identified in the search to the number of studies included in the review, ideally using a flow diagram. | Results + Figure 1 |
|  | 16b | Cite studies that might appear to meet the inclusion criteria, but which were excluded, and explain why they were excluded. | NR |
| Study characteristics | 17 | Cite each included study and present its characteristics. | Appendices C - G |
| Risk of bias in studies | 18 | Present assessments of risk of bias for each included study. | NR |
| Results of individual studies | 19 | For all outcomes, present, for each study: (a) summary statistics for each group (where appropriate) and (b) an effect estimate and its precision (e.g. confidence/credible interval), ideally using structured tables or plots. | NR |
| Results of syntheses | 20a | For each synthesis, briefly summarise the characteristics and risk of bias among contributing studies. | Results +  Appendices C - G |
|  | 20b | Present results of all statistical syntheses conducted. If meta-analysis was done, present for each the summary estimate and its precision (e.g. confidence/credible interval) and measures of statistical heterogeneity. If comparing groups, describe the direction of the effect. | NR |
|  | 20c | Present results of all investigations of possible causes of heterogeneity among study results. | NR |
|  | 20d | Present results of all sensitivity analyses conducted to assess the robustness of the synthesized results. | NR |
| Reporting biases | 21 | Present assessments of risk of bias due to missing results (arising from reporting biases) for each synthesis assessed. | NR |
| Certainty of evidence | 22 | Present assessments of certainty (or confidence) in the body of evidence for each outcome assessed. | NR |
| **DISCUSSION** | | |  |
| Discussion | 23a | Provide a general interpretation of the results in the context of other evidence. | Discussion  Para 2 – 5 |
|  | 23b | Discuss any limitations of the evidence included in the review. | Discussion  Para 6 |
|  | 23c | Discuss any limitations of the review processes used. | Discussion  Para 6 |
|  | 23d | Discuss implications of the results for practice, policy, and future research. | Discussion  Para 7 – 14 |
| **OTHER INFORMATION** | | |  |
| Registration and protocol | 24a | Provide registration information for the review, including register name and registration number, or state that the review was not registered. | NR |
|  | 24b | Indicate where the review protocol can be accessed, or state that a protocol was not prepared. | Supplementary material B |
|  | 24c | Describe and explain any amendments to information provided at registration or in the protocol. | NR |
| Support | 25 | Describe sources of financial or non-financial support for the review, and the role of the funders or sponsors in the review. | Title page |
| Competing interests | 26 | Declare any competing interests of review authors. | Title page |
| Availability of data, code and other materials | 27 | Report which of the following are publicly available and where they can be found: template data collection forms; data extracted from included studies; data used for all analyses; analytic code; any other materials used in the review. | NR |

*From:* Page MJ, McKenzie JE, Bossuyt PM, Boutron I, Hoffmann TC, Mulrow CD, et al. The PRISMA 2020 statement: an updated guideline for reporting systematic reviews. BMJ 2021;372:n71. doi: 10.1136/bmj.n71

# **Supplementary material B: Systematic review protocol**

*The protocol for the systematic review delineates the methodology, encompassing the review question and the search strategy.*

**Review Title:** Assessment of gender in suicide prevention in adolescents and young adults: a systematic review

**Review question:**

The purpose of this systematic review was to describe the current use of a gendered approach in AYA suicide prevention as follows: 1) to highlight how gender data are considered and translated when developing and implementing suicide prevention intervention and 2) to identify and describe gender-transformative approaches in suicide prevention programs.

**Search strategy:**

PubMed, Embase and APAPsycInfo were searched, using controlled vocabulary for each database and free text.

## Table B1. List of keywords used in the research

| **Suicide** | **Intervention** | **AJA** | **Gender** |
| --- | --- | --- | --- |
| Suicide  Suicid*  Non-fatal suicidal behavior  Suicidal behavior  Suicide attempt  Suicide attempt*  Parasuicide  Self-injury  Self-injur*  Self-injurious behavior  Self-harm  Self-harm*  Self-mutilation  Suicide ideation  Suicide plan  Suicidality | Intervention  Intervent*  Implementation  Implement*  Program  Program*  + Specific keywords on each database controlled vocabulory (Internet-based intervention ; Family intervention ; Crisis intervention ; …) | Adolescent  Adolescent*  Teenager  Teen*  Young Adult  Youth | Gender  Gender*  Sex  Female*  Male*  Woman/Women  Man/Men  Girl/Girls  Boy/Boys  Femininity  Masculinity  Gender norm*  Gender role*  Gender relation*  Gender-based violence  Sexism  Gender difference*  Gender identity  Sexual and gender minorit*  LGBTQ  LGBQT  LGBT*  Lesbian  Gay  Queer  Homosexual  Bisexual  Transgender  Nonbinary |

Selected articles had to have been accepted for publication between 2000 and August 2022. We limited the search to studies in French and English. In addition to our database, we included three previous systematic reviews on suicide prevention among AYA: Ougrin et al., 2015; Calear et al., 2016; Robinson et al., 2016.

*Eligibility criteria*

Studies were included if they examined interventions aimed at preventing suicidal behavior. Outcomes were not restricted and could include changes in suicide / suicidal behavior rates and trends, or changes in knowledge about suicidal behavior, among others. Studies were only included if the majority of the sample consisted of AYA between the ages of 12 and 25.

Exclusion criteria included articles where NSSI was the main focus or outcome, articles in which a risk factor for suicidal behavior was the main focus, and articles based on mental health, anxiety or depression. In addition, interventions that consisted solely of screening a population for suicidal risk or administering drug treatments were excluded, as were articles focused on the testing of new diagnostic tools and protocols. Interventions in adults, including pediatric health professionals or caregivers, were excluded.

The minimum criterion for gendered approach was to identify a sex or gender identity category for the non-mixed samples, or to distinguish between a minimum of two sex or gender identity categories for mixed samples. This criterion was only applied during the second step of the review, by reading the entire article.

*Selection process*

After the search, duplicates were removed and the articles were screened and assessed for eligibility by two reviewers (JK and LL).

*Data Collection process*

To ensure a double extraction of gender data from all eligible articles, one reviewer extracted data from all articles (LL) while the other two shared the database (VB and JK).

*Data items*

## Table B2. List of data items

| **Categories** | **Elements** |
| --- | --- |
| **Participants** | Type of participants  Study criteria  Characteristics at baseline |
| **Intervention** | Description  Setting  Preventive approach  Strategies |
| **Introduction** | Literature review and background  Research question(s) related to gender  Intervention setting related to gender |
| **Results** | Distribution, source of information and other demographics  Analysis details (quantitative/qualitative)  Reporting of findings (differences) |
| **Discussion** | Integration of gender-related findings in the conclusion  Explanation/hypothesis  Limitations/opening |
| **Use of language** | Vocabulary related to gender |
| **Gender approach** | Gender-blind, sensitive (minor/medium), specific, gender transformative elements |

*Study risk of bias assessment*

The quality of the studies was assessed using the Mixed-Method Appraisal Tool (MMA-T), version 2018. This tool facilitated the measurement of the quality in a variety of study designs, including qualitative studies, randomised controlled trials, non-randomised controlled trials, descriptive studies and mixed methods.

# **Supplementary material C: Quality appraisal**

The quality of the articles was assessed using the Mixed Methods Appraisal Tool (MMAT) – version 2018 (Hong et al., 2018). Our sample is predominantly composed of quantitative non-randomised studies. In accordance with the recommendations provided by Hong and colleagues, the calculation of a score was not performed.

The possible answers were “Yes”, “No” or “Can’t tell”. Cases with “Can’t tell” answers are of particular importance due to the paucity of detailed information in the articles.

There were no differences between designs related to the gender categories.

*From:* Hong QN, Fàbregues S, Bartlett G, Boardman F, Cargo M, Dagenais P, et al. The Mixed Methods Appraisal Tool (MMAT) version 2018 for information professionals and researchers. Educ Inf. 1 nov 2018;34(4):285‑91.

## Table C1: Qualitative studies (n = 5)

| **Reference** | **1.1** | **1.2** | **1.3** | **1.4** | **1.5** |
| --- | --- | --- | --- | --- | --- |
|  | **Qualitative approach appropriate** | **Qualitative data collection methods appropriate** | **Findings adequately derived** | **Interpretation sufficiently substantiated** | **Coherence** |
| Bailey *et al.*, 2021 Australia | Yes | Yes | Yes | Yes | Yes |
| Honea *et al.*, 2022 USA | Yes | Yes | Yes | Yes | Yes |
| Humensky, Gil,*et al.*, 2017 USA | Yes | Yes | Yes | Yes | Yes |
| Schiffler *et al.*, 2022 Austria | Yes | Yes | Yes | Yes | Yes |
| Tighe & McKay, 2012 Australia | Can't tell | Can't tell | Can't tell | Can't tell | Can't tell |

## Table C2: Quantitative randomised controlled trials (n = 56)

| **Reference** | **2.1** | **2.2** | **2.3** | **2.4** | **2.5** |
| --- | --- | --- | --- | --- | --- |
|  | **Appropriate randomisation** | **Comparable groups at baseline** | **Complete outcome data*** | **Outcome assessors blinded** | **Participants adherence to the intervention**** |
| Adrian, McCauley, *et al.*, 2019 USA | Yes | Yes | Yes | Yes | No |
| Alavi *et al.*, 2013 Iran | Can't tell | Yes | Yes | Can't tell | Can't tell |
| Anestis *et al.,* 2020 USA | Can't tell | No | No | Can't tell | Can't tell |
| Asarnow *et al.,* 2011 USA | Yes | Can't tell | Yes | Yes | Can't tell |
| Asarnow *et al.*, 2017 USA | Yes | Yes | No | Yes | Can't tell |
| Aseltine *et al.*, 2004 USA | Can't tell | No | Can't tell | Can't tell | Can't tell |
| Aseltine *et al.*, 2007 USA | Can't tell | Yes | Can't tell | Can't tell | Can't tell |
| Calear *et al.*, 2022 Australia | Yes | Yes | No | Can't tell | Can't tell |
| Cottrell *et al.*, 2018 UK | Yes | Yes | No | Can't tell | Can't tell |
| Czys *et al.*, 2019 USA | Yes | Can't tell | Yes | Yes | Yes |
| Czys et al., 2021 USA | Yes | Yes | Yes | Yes | Yes |
| Diamond *et al.,* 2010  USA - Israel | Yes | Yes | Yes | No | No |
| Dobias *et al.*, 2021 USA | Can't tell | Can't tell | No | Can't tell | No |
| Donaldson *et al.*, 2004 USA | Can't tell | Can't tell | Yes | Can't tell | Can't tell |
| Eggert *et al.*, 2002 USA | Yes | No | No | Can't tell | Can't tell |
| Eskin *et al.*, 2007 Turkey | Can't tell | No | Yes | Can't tell | Yes |
| Esposito-Smythers *et al.*, 2011 USA | Yes | Can't tell | No | No | No |
| Fitzpatrick *et al.*, 2005 USA | Can't tell | Can't tell | No | Can't tell | Can't tell |
| Green *et al.*, 2011 USA | Yes | Yes | Yes | Yes | No |
| Grupp-Phelan *et al.*, 2019 USA | Yes | Yes | Yes | Yes | Can't tell |
| Han *et al.,* 2018 Australia - China | Yes | No | No | Can't tell | Can't tell |
| Hazell *et al.*, 2009 Australia | Can't tell | No | No | Yes | No |
| Hetrick *et al.*, 2017 Australia | Yes | Can't tell | No | No | No |
| Hooven *et al.*, 2012 USA | Can't tell | No | Yes | Can't tell | Yes |
| Huey *et al.*, 2004 USA | Can't tell | Yes | Yes | Can't tell | Can't tell |
| Jegannathan *et al.*, 2014 Cambodia and Sweden | Can't tell | Can't tell | No | Can't tell | Can't tell |
| Kennard *et al.*, 2018 USA | Yes | No | No | Yes | Can't tell |
| King *et al.*, 2019 USA | Yes | Yes | No | Can't tell | Can't tell |
| King *et al.*, 2022 USA | Can't tell | Yes | No | Can't tell | Can't tell |
| King *et al.*, 2015 USA | Can't tell | Can't tell | No | Can't tell | Can't tell |
| King, Gipson, *et al.*, 2015 USA | Yes | Can't tell | Yes | Yes | Can't tell |
| King *et al.*, 2009 USA | Yes | Yes | No | Can't tell | Can't tell |
| King *et al.*, 2006 USA | Yes | No | No | No | Can't tell |
| Klim-Conforti *et al.*, 2021 Canada | No | Can't tell | No | No | Can't tell |
| Klimes-Dougan *et al.*, 2009 USA | Can't tell | Can't tell | NA | Can't tell | Can't tell |
| McManama O'Brien et al., 2018  USA | Yes | Yes | Yes | Can't tell | Yes |
| Mehlum *et al.*, 2018 Norway | Yes | Yes | Yes | Yes | Can't tell |
| Ougrin *et al.*, 2013 UK | Yes | Can't tell | Yes | No | Can't tell |
| Pineda & Dadds, 2013 Australia | Yes | Yes | No | Yes | Can't tell |
| Pistorello *et al.*, 2012  USA | Yes | Can't tell | No | Can't tell | Can't tell |
| Portzky & van Heeringen, 2006 Belgium | Can't tell | Yes | Yes | Can't tell | Can't tell |
| Randell *et al*.*,* 2001  USA | Yes | Can't tell | Yes | Can't tell | Can't tell |
| Robinson *et al.*, 2012 Australia | Yes | No | No | Yes | Can't tell |
| Robinson, Case, et al., 2016 USA | Can't tell | Can't tell | No | Can't tell | No |
| Rossouw & Fonagy, 2012 UK | Yes | Can't tell | Yes | Yes | Can't tell |
| Spirito *et al.*, 2002 USA | Can't tell | Yes | Yes | Can't tell | No |
| Spirito et al., 2015 USA | Can't tell | Can't tell | Yes | Can't tell | Can't tell |
| Tang *et al.*, 2009 Taiwan | Can't tell | Can't tell | Yes | Can't tell | No |
| Taylor-Rodgers & Batterham, 2014  Australia | Yes | Yes | Yes | No | Can't tell |
| Thompson *et al.*, 2001  USA | Yes | Can't tell | Yes | Can't tell | Can't tell |
| Wasserman *et al.*, 2015 Sweden (International) | Yes | Yes | No | Can't tell | Can't tell |
| Wharff *et al.*, 2019 USA | Yes | Can't tell | No | No | Can't tell |
| Wyman *et al.*, 2010 USA | Yes | Can't tell | No | Can't tell | Can't tell |
| Wilcox *et al.*, 2008 USA | Can't tell | Can't tell | Yes | Can't tell | Can't tell |
| Wood *et al.*, 2001 UK | Yes | Yes | Yes | Yes | Can't tell |
| Xavier *et al.*, 2019 Brazil | Yes | Yes | Yes | Yes | Yes |

** In accordance with the guidelines, the completion of the outcomes was to considered to have occurred when 80% of the sample had contributed to all outcomes, subsequent to randomisation and across both arms.*

** The study was considered to have adequate adherence if it was explicitly stated that at least 80% of the participants received all the components, attended all sessions, or completed all the modules. Adherence is a multifaceted concept that poses significant challenges in terms of measurement, often not addressed* (Giovanazzi et al., 2022)*.*

## Table C3: Quantitative non-randomised studies (n = 65)

| **Reference** | **3.1** | **3.2** | **3.3** | **3.4** | **3.5** |
| --- | --- | --- | --- | --- | --- |
|  | **Representative sample** | **Appropriate outcome measures** | **Complete outcome data** | **Confounders accounted for** | **Exposure occurred as intended** |
| Adrian, Blossom, *et al.*, 2021 USA | No | Yes | Yes | Can't tell | Yes |
| Allen *et al.*, 2018 USA | Yes | Yes | Can't tell | Can't tell | Can't tell |
| Asarnow *et al.,* 2015 USA | Yes | Yes | Yes | Can't tell | Can't tell |
| Babeva *et al.*, 2020 USA | Yes | Yes | Yes | Can't tell | Can't tell |
| Bailey *et al.*, 2020 Australia | Yes | Yes | Yes | Can't tell | Can't tell |
| Bailey *et al.*, 2017 Australia | Yes | Yes | No | Can't tell | Can't tell |
| Berk *et al.,* 2020  USA | Yes | Yes | No | Can't tell | Can't tell |
| Busby *et al.*, 2020 USA | Yes | Yes | Yes | Can't tell | Can't tell |
| Choi *et al.*, 2016 South Korea - USA | Yes | Yes | No | Can't tell | Can't tell |
| Ciffone, 2007 USA | Yes | Yes | Can't tell | Can't tell | Can't tell |
| Cloutier *et al.*, 2022 Canada | Yes | Yes | No | Can't tell | Can't tell |
| Courtney & Flament, 2015 Canada | Yes | Yes | No | Can't tell | Can't tell |
| Cwik *et al.*, 2016 USA | Yes | Yes | No | Can't tell | Can't tell |
| Diamond *et al.*, 2013  Israel - USA | Yes | Yes | Yes | Can't tell | Can't tell |
| Downs *et al.*, 2014 USA | Yes | Yes | NA | Can't tell | Can't tell |
| Duarté-Vélez *et al.*, 2016 USA | Yes | Yes | No | Can't tell | Can't tell |
| Esposito-Smythers *et al.*, 2006 USA | Yes | Yes | Yes | Can't tell | Can't tell |
| Flynn *et al.*, 2016 USA | Yes | Yes | Yes | Can't tell | Can't tell |
| Flyyn *et al.*, 2019 USA | Yes | Yes | Yes | Can't tell | Can't tell |
| Freedenthal, 2010 USA | Yes | Yes | No | Can't tell | Can't tell |
| Geddes *et al.*, 2013 Australia | Yes | Yes | Yes | Can't tell | Can't tell |
| Gillespie *et al.*, 2019 Ireland | Yes | Yes | No | Can't tell | Can't tell |
| Gryglewicz *et al.*, 2023 USA | Yes | Yes | No | Can't tell | Can't tell |
| Haas *et al.*, 2008 USA | Yes | Yes | No | Can't tell | Can't tell |
| Haruvi Catalan *et al.*, 2020 Spain | Yes | Yes | Yes | Can't tell | Can't tell |
| Hill *et al.*, 2020 USA | Yes | Yes | Yes | Can't tell | Can't tell |
| Hooven *et al.*, 2010 USA | Yes | Yes | No | Can't tell | Can't tell |
| Humensky, Coronel, *et al.*, 2017 USA | Yes | Yes | Can't tell | Can't tell | Can't tell |
| Hutcherson *et al.*, 2021 USA | Yes | Yes | Can't tell | Can't tell | Can't tell |
| James *et al.*, 2011  UK | Yes | Yes | No | Can't tell | Can't tell |
| James *et al.*, 2015 Germany and USA | Yes | Yes | No | Can't tell | Can't tell |
| Katz *et al.*, 2004 Canada | Yes | Yes | Yes | Can't tell | Can't tell |
| Kemp *et al.,* 2021 USA | Yes | Yes | Yes | Can't tell | Can't tell |
| Kennard et al., 2019 USA | Yes | Yes | Yes | Can't tell | Can't tell |
| Kim *et al.*, 2020 South Korea | Yes | Yes | No | Can't tell | Can't tell |
| King *et al.*, 2011 USA | Yes | Yes | No | Can't tell | Can't tell |
| King *et al.*, 2003 Australia | Yes | Yes | NA | Can't tell | Can't tell |
| Kohls *et al.*, 2022 Germany | Yes | Yes | No | Can't tell | Can't tell |
| La Sala *et al.*, 2021 Australia | Yes | Yes | No | Can't tell | Can't tell |
| Lindow *et al.*, 2020a USA | Yes | Yes | No | Can't tell | Yes |
| Lindow *et al.,* 2020b USA | Yes | Yes | No | Can't tell | Can't tell |
| Manjula *et al.*, 2018 India | Yes | Yes | Yes | Can't tell | Can't tell |
| McBee-Strayer *et al.*, 2019 USA | Yes | Yes | Yes | Can't tell | Can't tell |
| McGillivray *et al.*, 2021 Australia | Yes | Yes | No | Can't tell | Can't tell |
| McManama O'Brien *et al.*, 2017 USA | Yes | Yes | NA | Can't tell | Can't tell |
| Mohatt *et al.*, 2014 USA | Yes | Yes | No | Can't tell | Yes |
| Muela *et al.*, 2021 Spain | Yes | Yes | Yes | Can't tell | Can't tell |
| Negi & Sharma, 2020 India | Yes | Yes | Can't tell | Can't tell | Can't tell |
| Oldershaw *et al.*, 2012 UK | Yes | Yes | No | Can't tell | Can't tell |
| Pasco *et al.*, 2012 USA | Yes | Yes | No | Can't tell | Can't tell |
| Perera & Kathriarachchi, 2011 Sri Lanka | Yes | Yes | Yes | Can't tell | Can't tell |
| Pickering *et al.*, 2018 USA | Yes | Yes | No | Can't tell | Can't tell |
| Rathus & Miller, 2002 USA | Yes | Yes | No | Can't tell | Can't tell |
| Rengasamy & Sparks, 2019 USA | Yes | Yes | Can't tell | Can't tell | Can't tell |
| Robinson, Hetrick, *et al.*, 2016 Australia | Yes | Yes | No | Can't tell | Can't tell |
| Robinson *et al.*, 2015 Australia | Yes | Yes | No | Can't tell | Can't tell |
| Rotheram-Borus *et al.*, 2000 USA | Yes | Yes | Yes | Can't tell | Can't tell |
| Russon *et al.*, 2021 USA | Yes | Yes | Yes | Can't tell | Can't tell |
| Samuolis *et al.*, 2020 USA | Yes | Yes | Yes | Can't tell | Can't tell |
| Silk *et al.*, 2017 USA | Yes | Yes | NA | Can't tell | Can't tell |
| Silverstone *et al.*, 2015 Canada | Yes | Yes | No | Can't tell | Can't tell |
| Silverstone et al., 2017 Canada | Yes | Yes | No | Can't tell | Can't tell |
| Wharff *et al.*, 2012 USA | Yes | Yes | No | Can't tell | Can't tell |
| Wijana *et al.*, 2018 Sweden | Yes | Yes | Can't tell | Can't tell | Can't tell |
| Woodberry & Popenoe, 2008 USA | Yes | Yes | No | Can't tell | Can't tell |

* *The composition of our samples varied and expectations of representativeness could vary among samples (e.g., interventions in the care system or in schools). We considered a sample to be representative if it included adolescents and young adults between the ages of 12 and 25.*

** *We evaluated the relevance of the outcome measures based on the presence of at least one measure of suicidal behavior or thought.*

## Table C4: Quantitative descriptive studies (n = 1)

| **Reference** | **4.1** | **4.2** | **4.3** | **4.4** | **4.5** |
| --- | --- | --- | --- | --- | --- |
|  | **Adequate sampling strategy** | **Representative sample** | **Appropriate outcome measures** | **Low risk of nonresponse bias** | **Appropriate statistical analysis** |
| Salk et al., 2021  USA | Yes | Yes | Can't tell | Can't tell | Can't tell |

## Table C5: Mixed methods studies (n = 6)

| **Reference** | **5.1** | **5.2** | **5.3** | **5.4** | **5.5** |
| --- | --- | --- | --- | --- | --- |
|  | **Adequate rationale for mixed method** | **Appropriate components integration** | **Appropriate interpretation of qual and quant results** | **Divergences and inconsistences addressed** | **Adherence to quality criteria for each** |
| Arenas-Montreal *et al.*, 2020 Mexico | Can't tell | No | Yes | Can't tell | Can't tell |
| Broadbent & Papadopoulos, 2014 Australia | Yes | Can't tell | Can't tell | Can't tell | Can't tell |
| Calear *et al.*, 2021 Australia | Can't tell | Yes | Can't tell | Can't tell | Can't tell |
| Normand *et al.*, 2018 France | Yes | Yes | Can't tell | Can't tell | Can't tell |
| Pisani *et al.*, 2018 USA | Yes | Yes | Can't tell | Can't tell | Can't tell |
| Trinh & Goebert, 2020 USA | Yes | Yes | Can't tell | Can't tell | Can't tell |

# **Supplementary material D: Details on each studies by gender category**

## Table D1: Gender-blind studies (n = 65)

*The following table details the gender-blind studies, wherein the gendered approach is confined to the description of the sample.*

| **ID** | **Participants** | **Intervention** | **Preventive approach** | **Strategy/ies** | **Gender approach** | **Vocabulory** |
| --- | --- | --- | --- | --- | --- | --- |
| Adrian 2021 | **Participants:** 22 adolescents  **Age range:** 8 - 17 **Mean age:** 13,45  **Distribution:**  *sex assigned at birth*   - 68,2% of women - 31,8% of men - 0% of intersex people   *gender identity*   - 59,1% of women - 36,4% of men - 4,5% of transgender men - 0% of transgender women | **Name:** The Collaborative Assessment and Management of Suicide (CAMS) **Setting:** medical | Indicated | Management/Treatment | Gender-blind | *In the text:*  Girl   *In the table:*  Sex (assigned at birth) Female; male; intersex Gender Transgender female; transgender male |
| Alavi 2013 | **Participants**: 30 adolescents  **Age range:** 12 - 18 **Mean age:** 16,05  **Distribution:**   - 90% of women - 10% of men | **Name:** CBT Program **Setting:** medical | Indicated | Management/Treatment | Gender-blind | Sex (distribution) Female; male |
| Asarnow 2015 | **Participants:** 35 adolescents  **Age range:** 11 - 18 **Mean age:** 14,89  **Distribution:**   - 86% of women - 14% of men | **Name:** SAFETY program (Safe Alternatives for Teens & Youths) (CBT and DBT-informed family treatment) **Setting:** medical | Indicated | Management/Treatment | Gender-blind | Gender Female; male |
| Asarnow 2017 | **Participants:** 42 adolescents  **Age range:** 11 - 18 **Mean age:** 14,62  **Distribution:**   - 88,1% of women - 11,9% of men | **Name:** SAFETY program  **Setting:** medical | Indicated | Management/Treatment | Gender-blind | Gender Female; male Lesbian ; gay ; bisexual |
| Bailey 2020 | **Participants:** 20 adolescents and young adults **Age range:** 16 - 25 **Mean age:** 21,7  **Distribution:**   - 55% of women - 35% of men - 10% of transgender people | **Name:** "Affinity" (MOST Intervention or Moderated Online Social Therapy) **Setting:** medical | Indicated | Media, telephone and internet services  Management/Treatment  Peer-to-peer support | Gender-blind | Gender Female; male Transgender |
| Bailey 2021 | **Participants:** 15 adolescents and young adults **Age range:** 17 - 24 **Mean age:** 21,3  **Distribution:**   - 60% of women - 33,3% of men - 6,7% of transgender people | **Name:** "Affinity" (MOST Intervention or Moderated Online Social Therapy)  **Setting:** medical | Indicated | Media, telephone and internet services  Management/Treatment  Peer-to-peer support | Gender-blind | Gender Female; male Transgender |
| Cloutier 2022 | **Participants:** 46 adolescents  **Age range:** 13 - 17 **Mean age:** 14,4  **Distribution:**   - 91,3% of women - 2,2% of men - 6,5% of people in the category “other” | **Name:** BRAVA Intervention (DBT) **Setting:** medical | Indicated | Management/Treatment | Gender-blind | Gender Female; male  Other |
| Courtney 2015 | **Participants:** 61 adolescents  **Age range:** 15 - 18 **Mean age:** 16,5  **Distribution:**   - 93,4% of women - 6,6% of men | **Name:** A-DBT-A (adapted form of DBT for adolescents) **Setting:** medical | Indicated | Management/Treatment | Gender-blind | Gender  Female; male |
| Czyz 2019 | **Participants:** 36 adolescents **Age range:** 13 - 17  **Mean age:** 15,42  **Distribution:**   - 78,8% of women - 21,2% of men | **Name:** MI-SafeCope Intervention (MI)  **Setting:** medical | Indicated | Management/Treatment Contact-information/follow-up care Safety Planning | Gender-blind | Sex Female; male |
| Czyz 2021 | **Participants:** 80 adolescents  **Age range:** 13 - 17  **Mean age:** 15,6  **Distribution:**   - 67,5% of women - 32,5% of men | **Name:** MI-Enhanced Safety Plan (MI-SP)  **Setting:** medical | Indicated | Management/Treatment Contact-information/follow-up care | Gender-blind | Sex Female; male |
| Diamond 2010 | **Participants:** 66 adolescents  **Age range:** 12 - 17 **Mean age:** 15,19  **Distribution:**   - 86% of women - 14% of men | **Name:** Attachment-Based Family Therapy (ABFT)  **Setting:** medical | Indicated | Management/Treatment | Gender-blind | Gender Female; male |
| Donaldson 2004 | **Participants:** 39 adolescents  **Age range:** 12 - 17  **Mean age:** 15  **Distribution:**   - 82% of women - 18% of men | **Name:** Skills-based treatment (SBT) vs Supportive relationship treatment (SRT) **Setting:** medical | Indicated | Management/Treatment | Gender-blind | Gender Female; male  Woman; men |
| Downs 2014 | **Participants:** 341 adolescents and young adults  (medical students) **Age range:** / **Mean age:** 25,4  **Distribution:**   - 59,5% of women - 40,5% of men | **Name:** HEAR (Healer Education Assessment and Referral) program  **Setting:** school | Selective | Awareness and education Screening | Gender-blind | Gender Female; male |
| Eskin 2007 | **Participants:** 46 adolescents and young adults  **Age range:** / **Mean age:** 19,1  **Distribution:**   - 69,6% of women - 30,4% of men | **Name:** Problem-solving therapy **(**PST)  **Setting:** school | Indicated | Management/Treatment | Gender-blind | Sex Female, male |
| Esposito-Smythers 2011 | **Participants:** 40 adolescents  **Age range:** 13 - 17  **Mean age:** 15,7  **Distribution:**   - 68% of women - 32% of men | **Name:** I-CBT (*pilot study: see Esposito-Smythers 2006*) **Setting:** medical | Indicated | Management/Treatment | Gender-blind | Gender Female; male  Women |
| Esposito-Smythers 2006 | **Participants:** 6 adolescents  **Age range:** 14 - 16 **Mean age:** 15  **Distribution:**   - 83,3% of women - 16,7% of men | **Name:** Pilot study, I-CBT outpatient intervention  **Setting:** medical | Indicated | Management/Treatment | Gender-blind | Female, male  Gender *(once in a table)* |
| Fitzpatrick 2005 | **Participants:** 110 adolescents and young adults  **Age range:** 18 - 24  **Mean age:** 19,02  **Distribution:**   - 54,5% of women - 45,5% of men | **Name:** Brief Problem-Orientation intervention  **Setting:** medical | Indicated | Management/Treatment | Gender-blind | Female, male |
| Flynn 2019 | **Participants:** 84 adolescents  **Age range:** 13 - 18 **Mean age:** 15,72  **Distribution:**   - 85% of women - 15% of men | **Name:** DBT-A  **Setting:** medical | Indicated | Management/Treatment | Gender-blind | Female |
| Freedenthal 2010 | **Participants:** 146 adolescents  **Age range:** 14 - 18 **Mean age:** 15,8  **Distribution:**   - 59% of women - 41% of men | **Name:** Yellow Ribbon  **Setting:** school | Universal | Awareness and education School-based program | Gender-blind | Gender Female; male |
| Gillespie 2019 | **Participants:** 152 adolescents **Age range:** 13 - 18 **Mean age:** 15,67  **Distribution:**   - 85% of women - 15% of men | **Name:** DBT-A (16- VS 24-week programmes)  **Setting:** community / medical | Indicated | Management/Treatment | Gender-blind | Female |
| Haruvi Catalan 2020 | **Participants:** 26 adolescents  **Age range:** 9 - 17  **Mean age:** 13,44  **Distribution:**   - 61,5% of women - 38,5% of men | **Name:** Ultra-brief IPT-A based acute crisis-intervention **Setting:** medical | Indicated | Management/Treatment Safety planning Crisis intervention Contact-information/follow-up care | Gender-blind | Gender Female; male |
| Hazell 2009 | **Participants:** 72 adolescents  **Age range:** 12 - 16 **Mean age:** 14,49  **Distribution:**   - 90,3% of women - 9,7% of men | **Name:** Group intervention based on the principles of CBT, social skills training, ITP, group psychotherapy  **Setting:** medical | Indicated | Management/Treatment Care coordination | Gender-blind | Sex Female; male |
| Hetrick 2017 | **Participants:** 50 adolescents  **Age range:** 13 - 19  **Mean age:** 14,7  **Distribution:**   - 82% of women - 18% of men | **Name:** Reframe-IT intervention (CBT approach) **Setting:** school | Indicated | Management/Treatment  Media, telephone and internet services | Gender-blind | Gender (distribution) Female; male |
| Hill 2020 | **Participants:** 15 adolescents  **Age range:** 12 - 17  **Mean age:** 14,53   - 80% of women - 20% of men | **Name:** Safety Planning Assistant  **Setting:** medical | Indicated | Safety planning Media, telephone and internet services | Gender-blind | Female |
| Hooven 2012 | **Participants:** 615 adolescents  **Age range:** 14 - 19 **Mean age:** 16  **Distribution:**   - 60% of women - 40% of men | **Name:** Promoting CARE: 3 different brief suicide prevention protocols **Setting:** school | Indicated | Management/Treatment  Media, telephone and internet services | Gender-blind | Gender Female |
| Hutcherson 2021 | **Participants:** study 1 = pilot study; 29 adolescents study 2 = medical floor; 86 adolescents study 3 = CHRT-SR/SAFETY-A ; 218 adolescents  **Age range:** 10 - 17  **Mean age:** /  **Distribution:**  study 2   - 18,6% of women - 81,4% of men   study 3   - 76% of women - 24% of men | **Name:** SAFETY-A in emergency rooms and medical floors **Setting:** medical | Indicated | Management/Treatment Care coordination Screening | Gender-blind | Gender *(in the table only)* Girls; boys Female; male |
| James 2011 | **Participants:** 25 adolescents  **Age range:** 13 - 17 **Mean age:** 15,5  **Distribution:**   - 83,3% of women - 16,7% of men | **Name:** DBT treatment package  **Setting:** medical | Indicated | Management/Treatment Media, telephone and internet services Care coordination | Gender-blind | Gender Female; male Girl; boy |
| James 2015 | **Participants:** 99 adolescents  **Age range:** 12 - 18 **Mean age:** 14,86  **Distribution:**   - 84,8% of women - 15,2% of men | **Name:** DBT program **Setting:** medical | Indicated | Management/Treatment Media, telephone and internet services Care coordination | Gender-blind | Gender Female; male Girl; boy |
| Katz 2004 | **Participants:** 62 adolescents  **Age range:** 14 - 17  **Mean age:** 15,4  **Distribution:**   - 83,9% of women - 16,1% of men | **Name:** DBT program  **Setting:** medical | Indicated | Management/Treatment | Gender-blind | Females  Women Girls; boys |
| Kemp 2021 | **Participants:** 15 adolescents  **Age range:** 13 - 17 **Mean age:** 15,36  **Distribution:**   - 46,7% of women - 53,3% of men | **Name:** Screening and brief intervention for psychiatric and suicide risk in juvenile justice system  **Setting:** medical / institution | Indicated | Screening Safety planning Management/Treatment Care coordination | Gender-blind | (Biological) sex Female; male |
| King 2015 | **Participants:** 49 adolescents **Age range:** 14 - 19 **Mean age:** 17,7  **Distribution:**   - 56,1% of women - 43,9% of men | **Name:** Teen Options for Change (TOC)  **Setting:** medical | Indicated | Management/Treatment Safety planning  Crisis intervention | Gender-blind | Gender Female; male |
| King 2011 | **Participants:** 1030 adolescents  **Age range:** 14 - 18 **Mean age:** 14,1  **Distribution:**   - 56,1% of women - 43,9% of men | **Name:** Surviving the Teens Suicide Prevention and Depression Awareness Program  **Setting:** school | Universal | Awareness and education School-based program | Gender-blind | Sex Gender Female; male |
| King 2003 | **Participants:** 101 adolescents  **Age range:** < 18 **Mean age:** /  **Distribution:**  *(not all the sample, n = 73)*   - 79,5% of women - 20,5% of men | **Name:** Kids Help Line, telephone counselling service **Setting:** community | Indicated | Media, telephone and internet services  Management/Treatment | Gender-blind | Gender Female; male |
| Lindow 2020a | **Participants:** 436 adolescents  **Age range:** 12 - 16 **Mean age:** 14,5  **Distribution:**   - 61,3% of women - 38,7% of men - *With 6 participants reported “other” for gender* | **Name:** YAM (youth aware of mental health) adapted for 9th grade classrooms  **Setting:** school | Universal | School-based program  Awareness and education | Gender-blind | Female; male |
| Lindow 2020b | **Participants:** 436 adolescents  **Age range:** 12 - 18  **Mean age:** 14,5  **Distribution:**   - 61,3% of women - 38,7% of men - *With 6 participants reported “other” for gender* | **Name:** YAM (youth aware of mental health) adapted for 9th grade classrooms  **Setting:** school | Universal | School-based program  Awareness and education | Gender-blind | Female; male |
| McBee-Strayer 2019 | **Participants:** 50 adolescents  **Age range:** 12 - 17 **Mean age:** 15,1  **Distribution:**   - 86% of women - 14% of men | **Name:** Intensive crisis intervention (CBT, family-centered treatment)  **Setting:** medical | Indicated | Management/Treatment Crisis intervention | Gender-blind | Female |
| McGillivray 2021 | **Participants:** 556 adolescents  **Age range:** 13 - 16 **Mean age:** 14,4  **Distribution:**   - 56,6% of women - 43,4% of men | **Name:** YAM (youth aware of mental health) program  **Setting:** school | Universal | School-based program  Awareness and education | Gender-blind | Sex Female; male  Sexual orientation; LGBTQ+; heterosexual |
| McManama O'Brien 2017 | **Participants:** 20 adolescents  **Age range:** 13 - 18  **Mean age:** 15,7  **Distribution:**   - 80% of women - 20% of men | **Name:** Crisis Care (smartphone application, following discharge) **Setting:** medical | Indicated | Media, telephone and internet services Management/Treatment  Contact-information/follow-up care | Gender-blind | Gender  Female; male |
| McManama O'Brien 2018 | **Participants:** 50 adolescents  **Age range:** 14 - 17 **Mean age:** 15,8  **Distribution:**   - 80% of women - 20% of men - *With 1 transgender men and one person that declined this question* | **Name:** ASIST (Alcohol and Suicide Intervention for Suicidal Teens) (brief motivational enhacement intervention, MI) **Setting:** medical | Indicated | Management/Treatment | Gender-blind | Sex Gender Female; male  Transgender (FTM)  Sexual orientation; heterosexual; gay/lesbian/homosexual; bisexual) |
| Mehlum 2018 | **Participants:** 77 adolescents  **Age range:** 12 - 18  **Mean age:** 15,6  **Distribution:**   - 88,3% of women - 11,7% of men | **Name:** DBT-A  **Setting:** medical | Indicated | Management/Treatment | Gender-blind | Gender  Sex Female; male |
| Mohatt 2014 | **Participants:** 54 adolescents for the ET study ; 52 adolescents for the YA study  (Yup’ik Alaska Native Youth) **Age range:** 12 - 17 **Mean age:**  - ET study = 14,2  - YA study = 14,6  **Distribution:**  ET study   - 57,4% of women - 42,6% of men   YA study   - 48,1% of women - 51,9% of men | **Name:** The *Qungasvik*, a toolbox for community intervention (the Elluam Tungiinun and Yupiucimta Asvairtuumallerkaa Studies)  **Setting:** community | Selective | General public information  Awareness and education | Gender-blind | Gender Female; male |
| Oldershaw 2012 | **Participants:** 55 adolescents  **Age range:** 12 - 18  **Mean age:** /  **Distribution:**   - 85,5% of women - 14,5% of men | **Name:** CBT  **Setting:** medical | Indicated | Management/Treatment | Gender-blind | Gender Female; male |
| Ougrin 2013 | **Participants:** 70 adolescents  **Age range:** 12 - 18  **Mean age:** 15,55  **Distribution:**   - 80% of women - 20% of men | **Name:** Therapeutic Assessment  **Setting:** medical | Indicated | Management/Treatment | Gender-blind | Female |
| Perera 2011 | **Participants:** 124 adolescents and young adults  **Age range:** 15 - 24  **Mean age:** /  **Distribution:**   - 50,6% of women - 49,4% of men | **Name:** Problem-solving counseling  **Setting:** medical | Indicated | Management/Treatment | Gender-blind | Gender  Sex-ratio Female; male |
| Pineda 2013 | **Participants:** 48 adolescents  **Age range:** 12 - 17  **Mean age:** *(n = 40)* 15,13  **Distribution:**  *(n = 40)*   - 75% of women - 25% of men | **Name:** Resourceful Adolescent Parent Program  **Setting:** medical | Indicated | Management/Treatment Crisis intervention Safety planning | Gender-blind | Gender Female; male  Girl; boy |
| Pistorello 2012 | **Participants:** 63 adolescents and young adults  **Age range:** 18 - 25  **Mean age:** 20,86  **Distribution:**   - 81% of young women - 19% of young men | **Name:** DBT **Setting:** school | Indicated | Management/Treatment  School-based program | Gender-blind | Gender Female, male |
| Randell 2001 | **Participants:** 341 adolescents  **Age range:** 14 - 19 **Mean age:** /  **Distribution:**  *depending on the three conditions:*   - 48-59% of women - 41-52% of men | **Name:** C-CARE intervention + CAST (two brief interventions) **Setting:** school | Universal | General public information Awareness and education Management/Treatment | Gender-blind | Gender Female; male |
| Rathus 2002 | **Participants:** 111 adolescents **Age range:** / **Mean age:** 15,81  **Distribution:**   - 87,4% of women - 12,6% of men | **Name:** DBT  **Setting:** medical | Indicated | Management/Treatment | Gender-blind | Girl; boy Female Women |
| Robinson 2016a | **Participants:** 32 adolescents  **Age range:** 14 - 18 **Mean age:** 15,63  **Distribution:**   - 87,5% of women - 12,5% of men | **Name:** Reframe-IT intervention (CBT approach) **Setting:** school | Indicated | Media, telephone and internet services  Awareness and education Management/Treatment | Gender-blind | Gender *(in Table 1)* Female |
| Robinson 2015 | **Participants:** 32 adolescents  **Age range:** 14 - 18 **Mean age:** 15,63  **Distribution:**   - 87,5% of women - 12,5% of men | **Name:** Reframe-IT intervention (CBT approach) **Setting:** school | Indicated | Media, telephone and internet services  Awareness and education Management/Treatment | Gender-blind | Gender *(in Table 1)* Female |
| Robinson 2012 | **Participants:** 164 adolescents and young adults **Age range:** 15 - 24  **Mean age:** 18,6  **Distribution:**   - 64,6% of women - 35,4% of men | **Name:** Receipt of regular postcards  **Setting:** medical | Indicated | Media, telephone and internet services  Awareness and education  Contact-information/follow-up care | Gender-blind | Female; male |
| Rossouw 2012 | **Participants:** 80 adolescents **Age range:** 12 - 17 **Mean age:** 14,7  **Distribution:**   - 85% of women - 15% of men | **Name:** Mentalization-based treatment for adolescents (MBT-A) program  **Setting:** medical | Indicated | Management/Treatment | Gender-blind | Gender Female; male |
| Samuolis 2020 | **Participants:** 161 adolescents and young adults  **Age range:** 18 - 24 **Mean age:** 20  **Distribution:**   - 89% of women - 11% of men | **Name:** Question, Persuade, and Refer Gatekeeper Training for Suicide Prevention (QPR) **Setting:** school | Universal | Awareness and education Gatekeeper training Peer-to-peer support | Gender-blind | Gender Female; male Men |
| Schiffler 2022 | **Participants:** 13 adolescents and young adults **Age range:** 18 - 23 **Mean age:** 20,1  **Distribution:**   - 92,3% of women - 7,7% of men | **Name:** TalentLMS (DBT approach) **Setting:** medical | Indicated | Media, telephone and internet services  Awareness and education Management/Treatment | Gender-blind | Gender Female, male Women, man |
| Silk 2017 | **Participants:** 391 adolescents and young adults **Age range:** 18 - 28 **Mean age:** 19,01  **Distribution:**   - 60,6% of women - 39,4% of men | **Name:** Campus social norms campaign (peer and celebrity sources)  **Setting:** community / school | Universal | General public information Awareness and education | Gender-blind | Gender Female; male |
| Silverstone 2015 | **Participants:** 3244 adolescents  **Age range:** 10 - 19 **Mean age:** /  **Distribution:**   - 48,3% of women - 51,7% of men | **Name:** EMPATHY program: repeated data collection, identification of a high-risk group, CBT interventions **Setting:** school | Universal and Indicated | Media, telephone and internet services School-based program Management/Treatment Screening | Gender-blind | Sex Female; male |
| Silverstone 2017 | **Participants:** 6651 adolescents  **Age range:** 11 - 18 **Mean age:** /  **Distribution:**   - 34% of women - 32% of men - 34% of people declined to identify their gender identity | **Name:** EMPATHY program: repeated data collection, identification of a high-risk group, CBT interventions **Setting:** school | Universal | Media, telepgone and internet services School-based program Management/Treatment Screening | Gender-blind | Gender Female; male |
| Spirito 2002 | **Participants:** 63 adolescents **Age range:** 12 - 18  **Mean age:** /  **Distribution:**   - 90,5% of women - 9,5% of men | **Name:** Problem-solving intervention  **Setting:** medical | Indicated | Management/Treatment Care coordination | Gender-blind | Gender Female, male |
| Spirito 2015 | **Participants:** 24 adolescents **Age range:** 11 - 17  **Mean age:** 14,46  **Distribution:**   - 83,3% of women - 16,7% of men | **Name:** Adolescent-Only CBT and Parent-Adolescent CBT **Setting:** medical | Indicated | Management/Treatment | Gender-blind | Gender Female |
| Tang 2009 | **Participants:** 73 adolescents  **Age range:** 12 - 18  **Mean age:** 15,25  **Distribution:**   - 65,8% of women - 34,2% of men | **Name:** IPT-A-IN  **Setting:** school | Indicated | School-based program Management/Treatment | Gender-blind | Gender Sex Girl; boy |
| Taylor-Rodgers 2014 | **Participants:** 67 adolescents and young adults **Age range:** 18 - 25  **Mean age:** 21,9  **Distribution:**   - 74,6% of women - 25,4% of men | **Name:** Online psychoeducation intervention  **Setting:** community | Universal | General public information Media, telephone and internet services  Awareness and education Management/Treatment | Gender-blind | Gender Female; male |
| Trinh 2020 | **Participants:** 44 adolescents  **Age range:** /  **Mean age:** /  **Distribution:**   - 64% of women - 32% of men - 4% of participants who do not identify with their sex assigned at birth | **Name:** 2019 Prevent Suicide Hawai'i Conference: Hope, Help, and Healing (2-day train-the-trainer workshop, games and activities)  **Setting:** community | Universal | General public information Awareness and education Gatekeeper training Peer-to-peer support | Gender-blind | Gender  Sex (assigned at birth) Female; male; other |
| Wood 2001 | **Participants:** 63 adolescents **Age range:** 12 - 16  **Mean age:** 14,25  **Distribution:**   - 77,8% of women - 22,2% of men | **Name:** Group therapy  **Setting:** medical | Indicated | Management/Treatment Peer-to-peer support | Gender-blind | Female Boy |
| Woodberry 2008 | **Participants:** 46 adolescents  **Age range:** 13 - 18 **Mean age:** 16  **Distribution:**   - 89% of women - 11% of men | **Name:** DBT program **Setting:** community / medical | Indicated | Management/Treatment | Gender-blind | Gender Sex Girl  Female; male Women |
| Xavier 2019 | **Participants:** 100 adolescents **Age range:** 15 - 19 **Mean age:** 17,2  **Distribution:**   - 60% of women - 40% of men | **Name:** Problem-solving intervention  **Setting:** school / medical | Indicated | Awareness and Education Management/Treatment | Gender-blind | Sex Female, male Women |

*Notes: A-DBT-A: Adapted Dialectical Behavior Therapy for Adolescents; CBT: Cognitive-behavioral therapy; DBT: Dialectical behavior therapy; I-CBT: Integrative cognitive behavioral therapy; IPT-A: Interpersonal psychotherapy for adolescents; MI: Motivational Interviewin*

## Table D2: Studies with minor sensitivity to gender (n = 20)

*The following table details the studies with a minor sensitivity to gender, wherein the gendered approach is problematised, yet the extant results and analysis are limited.*

| **ID** | **Participants** | **Intervention** | **Preventive approach** | **Strategy/ies** | **Gender approach** | **Vocabulory** |
| --- | --- | --- | --- | --- | --- | --- |
| Adrian 2019 | **Participants:** 173 adolescents  **Age range:** /  **Mean age:** 14,89  **Distribution:**   - 95% women - 5% men | **Name:** DBT or IGST  **Setting:** medical | Indicated | Management/Treatment | Gender sensitive (minor) | Sex  Female; male |
| Asarnow 2011 | **Participants:** 181 adolescents **Age range:** 10 - 18 **Mean age:** 14,7  **Distribution:**   - 69% women - 31% men | **Name:** Family Intervention for Suicide Prevention (FISP)  **Setting:** medical | Indicated | Management/Treatment | Gender sensitive (minor) | *In the table:* Sex Female; male   *In the text:*  Gender |
| Bailey 2017 | **Participants:** 129 adolescents **Age range:** 16 - 17 **Mean age:** 16,7  **Distribution:**   - 46,5% women - 53,5% men | **Name:** safeTALK  **Setting:** school | Universal | Awareness and Education School-based program Gatekeeper training  Screening | Gender sensitive (minor) | Gender Male |
| Calear 2022 | **Participants:** 1633 adolescents  **Age range:** 11 - 17 **Mean age:** 13,3  **Distribution:**   - 58,2% of women - 41,8% of men - *With participants that reported “other” for gender* | **Name:** Sources of Strength suicide prevention program **Setting:** school | Universal | Awareness and Education School-based program  Peer-to-peer support | Gender sensitive (minor) | Gender Sex Female; male  Other |
| Ciffone 2007 | **Participants:** 421 adolescents **Age range:** 15 - 16  **Mean age:** /  **Distribution:**   - 53% of women - 47% of men | **Name:** Curriculum-based high school program **Setting:** school | Universal | Awareness and Education School-based program | Gender sensitive (minor) | Gender Female; male |
| Cottrell 2018 | **Participants:** 832 adolescents  **Age range:** 11 - 17  **Mean age:** /  **Distribution:**   - 89% of women - 11% of men | **Name:** Self-Harm Intervention family therapy (SHIFT)  **Setting:** medical | Indicated | Management/Treatment | Gender sensitive (minor) | Sex  Female; male |
| Eggert 2002 | **Participants:** 341 adolescents **Age range:** 14 - 19 **Mean age:** 15,66  **Distribution:**   - 52% of women - 48% of men | **Name:** CAST (Coping And Support Training) and C-CAST (Counselors-CARE)  **Setting:** school | Indicated | Awareness and education School-based program  Screening | Gender sensitive (minor) | Gender Female; male |
| Green 2011 | **Participants:** 364 adolescents  **Age range:** 12 - 17  **Mean age:** /  **Distribution:**   - 89% of women - 11% of men | **Name:** ASSIST (Assessment of Treatment In Suicidal Teenagers)  **Setting:** medical | Indicated | Management/Treatment | Gender sensitive (minor) | Sex Male |
| Grupp-Phelan 2019 | **Participants:** 159 adolescents **Age range:** 12 - 17 **Mean age:** 15  **Distribution:**   - 79,2% of women - 20,8% of men | **Name:** Suicidal Teens Accessing Treatment After an Emergency Department Visit (STAT-ED)  **Setting:** medical | Indicated | Management/Treatment | Gender sensitive (minor) | Sex Female; male |
| Gryglewicz 2021 | **Participants:** 1125 adolescents  **Age range:** 10 - 17 **Mean age:** 14,6  **Distribution:**   - 75,2% of women - 24,8% of men | **Name:** LINC intervention **Setting:** medical | Indicated | Management/Treatment Contact-information/follow-up care Media, telephone and internet services | Gender sensitive (minor) | Female Heterosexual |
| Han 2018 | **Participants:** 257 adolescents and young adults (156 Chinese university students / 101 Australian university students)  **Age range:** 18- 30  **Mean age:**  Chinese students = 18,57  Australian students = 20,07  **Distribution:**  *Chinese students*   - 50% of women - 50% of men   *Australian students*   - 80,2% of women - 19,8% of men | **Name:** ProHelp program (online psychoeducational intervention)  **Setting:** school | Universal | Management/Treatment  Media, telephone and internet services | Gender sensitive (minor) | Sex Female; male |
| Hooven 2010 | **Participants:** 615 adolescents **Age range:** 14 - 18 **Mean age:** 15,9  **Distribution:**   - 60% of women - 40% of men | **Name:** Promoting CARE: 3 different brief suicide prevention protocols **Setting:** school | Indicated | Management/Treatment  Media, telephone and internet services | Gender sensitive (minor) | Gender  Sex Female |
| Kennard 2018 | **Participants:** 66 adolescents  **Age range:** 12 - 18  **Mean age:** 15,1  **Distribution:**   - 89,4% of women - 10,6% of men | **Name:** Brief app-supported inpatient intervention As Safe As Possible (ASAP)  **Setting:** medical | Indicated | Safety planning Media, telephone and internet services  Management/Treatment  Care coordination | Gender sensitive (minor) | Sex Female; male |
| Normand 2017 | **Participants:** 173 adolescents  **Age range:** 14 - 21 **Mean age:** 19,1  **Distribution:**   - 86,1% of women - 13,9% of men | **Name:** 4-phones-class procedure after discharge  **Setting:** medical | Indicated | Management/Treatment Care coordination Contact-information/follow-up care | Gender sensitive (minor) | Gender(/s)  Female; male  Girl; boy |
| Pasco 2012 | **Participants:** 65 adolescents and young adults **Age range:** 18 – 20+ **Mean age:** 19  **Distribution:**  *Missing data reported*   - 53,8% of women - 43,1% of men | **Name:** Campus Connect, gatekeeper-training program **Setting:** school | Universal | General public information Gatekeeper training  Peer-to-peer support | Gender sensitive (minor) | Gender  Sex  Female; male  Women; men |
| Rengasamy 2019 | **Participants:** 142 adolescents  **Age range:** / **Mean age:** 15  **Distribution:**   - 70% of women - 30% of men | **Name:** Single call intervention (SCI) or Multiple calls intervention (MCI)  **Setting:** medical | Indicated | Media, telephone and internet services Means restriction  Safety planning  Contact-information/follow-up care Screening | Gender sensitive (minor) | Gender (biological gender ; patient-identified gender)  Female; male |
| Wasserman 2015 | **Participants:** 11 110 adolescents (in different countries) **Age range:** 14 - 16 **Mean age:** 14,8  **Distribution:**   - 59% of women - 41% of men | **Name:** SEYLE (the Saving and Empowering Young Lives in Europe), three interventions:  - Question, Persuade, and Refer (QPR)  - The Youth Aware of Mental Health Program (YAM)  - The Screening by Professionnals (ProfScreen)  **Setting:** school | Universal | Awareness and education School-based program Screening Peer-to-peer support | Gender sensitive (minor) | Sex Girl |
| Wharff 2019 | **Participants:** 142 adolescents **Age range:** 13 - 18  **Mean age:** 15,5  **Distribution:**   - 72% of women - 28% of men | **Name:** FBCI  **Setting:** medical | Indicated | Management/Treatment Crisis intervention  Care coordination | Gender sensitive (minor) | Gender Female ; male |
| Wharff 2012 | **Participants:** 250 adolescents  **Age range:** 13 - 18  **Mean age:** 15,6  **Distribution:**   - 74,8% of women - 25,2% of men | **Name:** FBCI  **Setting:** medical | Indicated | Management/Treatment Crisis intervention  Care coordination | Gender sensitive (minor) | Gender Female ; male |
| Wyman 2010 | **Participants:** 3128 students  **Age range:** / **Mean age:** /  **Distribution:**   - 54% of women - 46% of men | **Name:** Sources of Strength suicide prevention program  **Setting:** school | Universal | Awareness and education Gatekeeper training School-based program  Peer-to-peer support | Gender sensitive (minor) | Gender Sex Female |

*Notes: DBT: Dialectical behavior therapy; IGST: Individual/Group supportive therapy*

## Table D3: Studies with medium sensitivity to gender (n = 34)

*The following table details the studies with a medium sensitivity to gender, wherein the gendered approach is problematised and there are a few results and hypotheses concerning gender differences.*

| **ID** | **Participants** | **Intervention** | **Preventive approach** | **Strategy/ies** | **Gender approach** | **Vocabulory** |
| --- | --- | --- | --- | --- | --- | --- |
| Allen 2018 | **Participants:** 120 adolescents  (Alaska Native youth) **Age range:** 12 - 17 **Mean age:** 14,46  **Distribution:**   - 39,8% of women - 60,2% of men | **Name:** The Qungasvik intervention  **Setting:** community | Selective | Awareness and education | Gender sensitivity (major) | Gender Female; male  Women, men |
| Arenas-Monreal 2020 | **Participants:** 29 adolescents in the educational intervention: 29 adolescents, from school ; 20 adolescents in the community intervention **Age range:** 10 - 19 **Mean age:**  Educational intervention = 12  Community intervention = 14  **Distribution:**  *Educational intervention*   - 55,2% of women - 44,8% of women   *Community intervention*   - 25% of women - 75% of men | **Name:** Educational and gatekeeper interventions  **Setting:** school / community | Universal | Screening Awareness and education Gatekeeper training  School-based program | Gender sensitivity (major) | Sex Female; male  Women; men |
| Aseltine 2004 | **Participants:** 2100 adolescents  **Age range:** 14-18 **Mean age:** /  **Distribution:**  *n = 2 041*   - 52,7% of women - 47,3% of men | **Name:** Signs of Suicide or SOS Program  **Setting:** school | Universal | Awareness and education School-based program  Screening  Peer-to-peer support | Gender sensitivity (major) | Gender Female; male  Girl; boy |
| Aseltine 2007 | **Participants:** 4133 adolescents **Age range:** 14-18 **Mean age:** /  *n = 4052*   - 50% of women - 50% of women | **Name:** Signs of Suicide or SOS Program  **Setting:** school | Universal | Awareness and education School-based program  Screening  Peer-to-peer support | Gender sensitivity (major) | Gender Sex Female; male Girl; boy |
| Babeva 2020 | **Participants:** 50 adolescents  **Age range:** 11-18  **Mean age:** 15,19  **Distribution:**   - 90% of women - 10% of men | **Name:** SAFETY Program  **Setting:** medical | Indicated | Management/Treatment | Gender sensitivity (major) | Gender Sex *(once in table 1)* Female; male Girls Sexual preference Non-heterosexual orientation |
| Berk 2020 | **Participants:** 24 adolescents  **Age range:** 12-17 **Mean age:** 15,2  **Distribution:**   - 92% of women - 8% of men | **Name:** DBT program **Setting:** medical | Indicated | Management/Treatment | Gender sensitivity (major) | Gender Female; male |
| Busby 2020 | **Participants:** 234 adolescents  **Age range:** 12 - 18 **Mean age:** 15,3  **Distribution:**   - 83% of women - 17% of men | **Name:** ED-STARS (Telephone hotline service) **Setting:** medical | Indicated | Contact-information/follow-up care Media, telephone and internet services | Gender sensitivity (major) | Gender Female; male |
| Choi 2016 | **Participants:** 431 adolescents and young adults  **Age range:** 17 - 30+ **Mean age:** 23,2  **Distribution**   - 73,5% of women - 26,5% of men | **Name:** Reaching Out, Lifting Up, web-based suicide awareness programme **Setting:** school | Universal | Media, telephone and internet services | Gender sensitivity (major) | Gender Female; male |
| Cwik 2016 | **Participants:** 13 adolescents  **Age range:** 10 - 19 **Mean age:** 14,3  **Distribution:**   - 92% of women - 8% of men | **Name:** New Hope, the Rotheram-Borus intervention for American Indian adolescents  **Setting:** community | Indicated | Awareness and Education Management/Treatment | Gender sensitivity (major) | Female; male |
| Flynn 2016 | **Participants:** 3 257 adolescents **Age range:** 11 - 18  **Mean age:** /  **Distribution:**   - 51,3% of women - 48,7% of men | **Name:** Yellow Ribbon **Setting:** school | Universal | Awareness and education School-based program Media, telephone and internet services | Gender sensitivity (major) | Gender Sex Female; male  Gender role theory |
| Haas 2008 | **Participants:** 1 162 adolescents **Age range:** / **Mean age:** /  **Distribution:**   - 71,8% of women - 28,2% of men | **Name:** Interactive web-based method of outreach **Setting:** school | Indicated | Screening Management/Treatment Media, telephone and internet services | Gender sensitivity (major) | Sex Female; male  Women; men |
| Huey 2003 | **Participants:** 156 adolescents  **Age range:** 10 - 17 **Mean age:** 12,9  **Distribution:**   - 35% of women - 65% of men | **Name:** Multisystematic therapy (MST) **Setting:** medical | Indicated | Management/Treatment Care coordination | Gender sensitivity (major) | Gender Female; male |
| Jegannathan 2014 | **Participants:** 299 students  **Age range:** / **Mean age:** /  **Distribution:**   - 51,5% of women - 48,5% of men | **Name:** Universal skill-training program  **Setting:** school | Universal | Awareness and education School-based program | Gender sensitivity (major) | Gender Gender difference Girl; boy Female; male |
| Kennard 2019 | **Participants:** 394 adolescents  **Age range:** 12 - 18 **Mean age:** 14,90  **Distribution:**   - 89,4% of women - 10,6% of men | **Name:** Intensive outpatient program (including CBT and DBT approaches) **Setting:** medical | Indicated | Safety Planning Management/Treatment Care coordination  Contact-information/follow-up care | Gender sensitivity (major) | Gender Gender dysphoria *(as a clinical characteristic)* Female; male |
| Kim 2020 | **Participants:** 768 adolescents  **Age range:** 12 - 18 **Mean age:** /  **Distribution:**   - 44,1% of women - 55,9% of men | **Name:** School-based mental health education program  **Setting:** school | Universal | School-based program  Awareness and education | Gender sensitivity (major) | Gender Female; male |
| King 2019 | **Participants:** 448 adolescents **Age range:** 13 - 17 *(analyses conducted on follow-up 11 to 14 years after)*  **Mean age:** 15,6  **Distribution:**   - 71,2% of women - 28,8% of men | **Name:** Youth-Nominated Support Team Intervention (psychoeducational social support intervention involving adults) Version II (YST-II) **Setting:** medical | Indicated | Management/Treatment Care coordination | Gender sensitivity (major) | Sex Female; male |
| King 2022 | **Participants:** 3 363 adolescents and young adults  **Age range:** 18 - 31+ **Mean age:** /  **Distribution:**   - 62,2% of young women - 35% of young men - 2,8% of transgender/genderqueer young people | **Name:** eBridge (Electronic Bridge to Mental Health for College Students) (MI principles)  **Setting:** school | Indicated | Media, telephone and internet services  School-based program Management/Treatment Care coordination Screening | Gender sensitivity (major) | Sex Gender Transgender / Genderqueer Female; male  Women; men |
| King 2015 | **Participants:** 76 adolescents and young adults **Age range:** / **Mean age:** 22,9  **Distribution**   - 59,21% of women - 40,79% of men | **Name:** eBridge (Electronic Bridge to Mental Health for College Students) (MI principles) **Setting:** school | Indicated | Media, telephone and internet services  School-based program Management/Treatment Care coordination Screening | Gender sensitivity (major) | Sex Gender Female; male Women; men |
| King 2009 | **Participants:** 448 adolescents  **Age range:** 13 - 17 **Mean age:** 15,65  **Distribution:**   - 71,2% of women - 28,8% of men | **Name:** Youth-Nominated Support Teamt Intervention (psychoeducational social support intervention involving adults) Version II (YST-II)  **Setting:** medical | Indicated | Management/Treatment Care coordination | Gender sensitivity (major) | Gender Sex Female: male Girl; boy |
| King 2006 | **Participants:** 289 adolescents  **Age range:** 12 - 17 **Mean age:** 15,3  **Distribution:**   - 68,2% of women - 31,8% of men | **Name:** Youth-Nominated Support Teamt Intervention (psychoeducational social support intervention involving adults) Version 1 (YST-1) **Setting:** medical | Indicated | Management/Treatment Care coordination | Gender sensitivity (major) | Gender Sex Female; male Girl; boy |
| Klim-Conforti 2021 | **Participants:** 430 adolescents  **Age range:** 11 - 14  **Mean age:** /  **Distribution:**   - 61,7% of women - 38,1% of men - 0,2% of non-binary people | **Name:** Harry Potter-based mental health literacy curriculum (CBT approach)  **Setting:** schools | Universal | Awareness and education School-based program | Gender sensitivity (major) | Sex Female; male Girl; boy Non-binary |
| Klimes-Dougan 2009 | **Participants:** 426 adolescents **Age range:** 14 - 18  **Mean age:** 15,24  **Distribution:**   - 56% of women - 44% of men | **Name:** Public service announcements  **Setting:** school / community | Universal | General public information Awareness and education | Gender sensitivity (major) | Gender  Female; male |
| Kohls 2022 | **Participants:** 6 962 adolescents and young adults  **Age range:** 7 - 25  **Mean age:** /  **Distribution:**  *For those who disclosed their gender (n = 1262)*   - 84,4% of young women - 13,1% of young men - 2,5% of diverse people | **Name:** Krisenchat, messenger-based chat counseling service **Setting:** community | Indicated | Media, telephone and internet services Management/Treatment  Care coordination | Gender sensitivity (major) | Gender Girl; boy Women; men Female; male; gender diverse LGBTQIA+ |
| La Sala 2021 | **Participants:** 189 adolescents and young adults **Age range:** 16 - 25  **Mean age:** 18,37  **Distribution:**   - 67,8% of women - 25,9% of men - 2,6% of transgender people - 2,6% of gender fluid people - 1,1% of gender neutral people | **Name:** Social medial intervention, #chatsafe campaign **Setting:** community | Universal | General public information Media, telephone and internet services  Awareness and education | Gender sensitivity (major) | Gender Gender identity Gender fluid Transgender Gender neutral Female; male Sexual orientation  Heterosexual ; Gay ; Bisexual ; Questioning ; non-heterosexual |
| Manjula 2018 | **Participants:** 1428 adolescents  **Age range:** /  **Mean age:** 15,35  **Distribution:**  *On the post assessment sample (n = 379)*   - 54% of women - 46% of men | **Name:** Brief universal school-based awareness program (CBT approach) **Setting:** school | Universal | School-based program  Awareness and education | Gender sensitivity (major) | Gender Girl; boy Female; male |
| Muela 2021 | **Participants:** 30 adolescents  **Age range:** 14 - 17  **Mean age:** 15,5  **Distribution:**   - 63,3% of women - 36,7% of men | **Name:** the OverComme-AAI program (animal-assisted intervention)  **Setting:** medical | Indicated | Management/Treatment | Gender sensitivity (major) | Genders  Sex; sexes  Women; men  Females; males  Girls; boys |
| Negi 2020 | **Participants:** 60 adolescents  **Age range:** 13 - 18  **Mean age:** /  **Distribution:**   - 50% of women - 50% of men | **Name:** Interpersonal therapy sessions  **Setting:** school | Indicated | School-based program Management/Treatment | Gender sensitivity (major) | Gender Female; male Girl; boy Women; men |
| Pickering 2018 | **Participants:** 5677 adolescents  **Age range:** 14 - 18  **Mean age:** /  **Distribution:**  *Missing data reported*   - 48,6% of young women - 49,9% of young men | **Name:** Sources of Strength suicide prevention program **Setting:** school | Universal | Awareness and education School-based program  Gatekeeper training  Peer-to-peer support | Gender sensitivity (major) | Gender  Sex Female; male |
| Pisani 2018 | **Participants:** 42 adolescents  **Age range:** 14 - 15  **Mean age:** /  **Distribution:**   - 71,4% of women - 28,6% of men | **Name:** Text4Strength (Sources of Strength extension)  **Setting:** school | Universal | Media, telephone and internet services Awareness and education School-based program Peer-to-peer support | Gender sensitivity (major) | Sex Female, male Girl, boy |
| Portzky 2006 | **Participants:** 172 adolescents **Age range:** 14 - 18  **Mean age:** 15,6  **Distribution**:   - 62,7% of women - 37,3% of men | **Name:** Psycho-educational program  **Setting:** school | Universal | Awareness and education School-based program Management/Treatment  Peer-to-peer support | Gender sensitivity (major) | Gender Female; male Girl; boy |
| Robinson 2016b | **Participants:** 758 adolescents  **Age range:** 15 - 17  **Mean age:** /  **Distribution:**   - 60% of women - 40% of men | **Name:** the A-CWS (Adapted-Coping With Stress course)  **Setting:** school | Selective | Awareness and education  School-based program  Management/Treatment  Screening Peer-to-peer support | Gender sensitivity (major) | Gender Female; male |
| Thompson 2001 | **Participants:** 460 adolescents  **Age range:** 14 - 19  **Mean age:** /   - 52% of women - 48% of men | **Name:** C-CARE intervention + CAST (two brief interventions) **Setting:** school | Indicated | Awareness and education School-based program Management/Treatment  Peer-to-peer support | Gender sensitivity (major) | Sex Female; male |
| Wijana 2018 | **Participants:** 49 adolescents  **Age range:** 13 - 19  **Mean age:** 14,6  **Distribution:**   - 85,7% of women - 14,3% of men | **Name:** Intensive Contextual Treatment (ICT) for Self-Harm **Setting:** medical | Indicated | Management/Treatment | Gender sensitivity (major) | Gender Female; male Girl; boy Women |
| Wilcox 2008 | **Participants:**  At baseline: 1196 children in Cohort 1 and 1115 in Cohort 2  At follow-up (15 years after first grade):   - Cohort 1 = 858 adolescents and young adults - Cohort 2 = 837 adolescents and young adults   **Age range:** (at follow-up)   - Cohort 1 = 20 - 24 - Cohort 2 = 19 – 23   **Mean age:** (at follow-up)   - Cohort 1 = 22 - Cohort 2 = 21   **Distribution:**  *Cohort 1, as young adults*   - 55% of women - 45% of men   *Cohort 2, as young adults*   - 52% of women - 48% of men | **Name:** The Good Behavior Game (GBG) vs Mastery Learning (ML) **Setting:** school | Universal | Awareness and education School-based program | Gender sensitivity (major) | Gender Sex Female; male Girl; boy |

*Notes: CBT: Cognitive-behavioral therapy; DBT: Dialectical behavior therapy; MI: Motivational Interviewing*

## Table D4: Studies with a gender specific approach (n = 11)

*The following table details the studies with a gender specific approach, wherein the gendered approach is examined through a focus on specific categories, including young women, men, or LGBTI+ individuals.*

| **ID** | **Participants** | **Intervention** | **Preventive approach** | **Strategy/ies** | **Gender approach** | **Vocabulory** |
| --- | --- | --- | --- | --- | --- | --- |
| Anestis 2020 | **Participants:** 73 adolescents  **Age range:** 16 - 18  **Mean age:** 16,71  **Distribution/Focus:**  100% of men | **Name:** DBT-A group session  **Setting:** military/school | Selective | Management/Treatment | Gender specific | Female; male  Girl; boy  Women; men  Gender differences  Mixed-gender sample |
| Broadbent 2014 | **Participants:** 119 adolescents and young adults **Age range:** 18 - 22 **Mean age:** *median age,* 20  **Distribution/Focus:**  100% of men | **Name:** Life Care Skills Programme **Setting:** work | Selective | Awareness and Education Peer-to-peer support | Gender specific | Female; male  Boy  Men; women |
| Calear 2021 | **Participants:** 594 adolescents  **Age range:** 16 - 18 **Mean age:** /  **Distribution/Focus:**  100% of men | **Name:** Silence is deadly  **Setting:** school | Selective | Awareness and Education School-based program Gatekeeper training | Gender specific | Gender Female; male  Gender roles, gender role conflict  Gender-based attitudes  Gender norms |
| Diamond 2013 | **Participants:** 10 adolescents **Age range:** 14 - 18 **Mean age:** 15,10  **Distribution/Focus:**  100% LGB people   - 80% of women - 20% of men | **Name:** Attachment-Based Family Therapy (ABFT)-LGB  **Setting:** medical | Indicated | Management/Treatment | Gender specific | Female; male  Gender atypical behavior Same-sex orientation  Minority sexual orientation  LGB  Lesbien; gay; bisexual |
| Dobias 2021 | **Participants:** 565 adolescents  **Age range:** 13 - 16  **Mean age:** 14,95  **Distribution/Focus:**  100% LGBTQ+ people  *n ≠ 100%*   - 66.4% of women - 8.7% of men - 8% of transgender people - 6% of Female to male transgender people (FTM) - 5,5% of Trans male people - 5,5% of Trans masculine people - 0,4% of Trans feminine people - 5,8% of Genderqueer people - 2,3% of Gender expansive people - 0,4% of Intersex people - 6,9% of Androgynous people - 19,3% of Nonbinary people - 1,1% of Two-spirited - 0,7% of Third gender people - 4,1% of Agender people - 10,6% of Not sure people - 4,6% of Other/Not Listed gender identities - 2,5% of Missing data | **Name:** Project SAVE (Stopping Adolescent Violence Everywhere) SSI **Setting:** community | Indicated | Media, telephone and internet services Management/Treatment | Gender specific | Gender identity: girl, boy ; woman, man ; female, male ; transgender, FTM, trans male, trans masculine, trans feminine, genderqueer, gender expansive, intersex, androgynous, nonbinary, two-spirited, third gender, agender, not sure, other/not listed, missing  Cis-gender Gender-diverse youth |
| Geddes 2013 | **Participants:** 6 adolescents  **Age range:** 14,6 - 15,7  **Mean age:** 15,1  **Distribution/Focus:**  100% of women | **Name:** DBT-A  **Setting:** medical | Indicated | Management/Treatment | Gender specific | Female Women Girls |
| Humensky 2017a | **Participants:** 105 adolescents  **Age range:** 11 - 18 **Mean age:** 14,9  **Distribution/Focus:**  100% of women | **Name:** Life is Precious (comprehensive services for mental health treatment, school and family support)  **Setting:** medical / community | Selective and Indicated | Management/Treatment Awareness and education Care coordination | Gender specific | Gender Gender roles  Female Girl |
| Humensky 2017b | **Participants:** 31 adolescents  **Age range:** / **Mean age:** /  **Distribution/Focus:**  100% of women | **Name:** Life is Precious (comprehensive services for mental health treatment, school and family support) **Setting:** medical / community | Selective and Indicated | Management/Treatment Awareness and education Care coordination | Gender specific | Gender Gender roles  Female; male Girl; boy |
| Rotheram-Borus 2000 | **Participants:** 140 adolescents **Age range:** 12 - 18 **Mean age:** 14,9  **Distribution/Focus:**  100% of women | **Name:** 18-Month Emergency Room Intervention  **Setting:** medical | Indicated | Management/Treatment Contact-information/Follow-up care | Gender specific | Female; male |
| Russon 2021 | **Participants:** 10 adolescents and young adults  **Age range:** 15 - 25 **Mean age:** 18,2  **Distribution:**  100% LGBTQ+ people | **Name:** Attachment-Based Family Therapy (ABFT)-LGBTQ+  **Setting:** medical | Indicated | Management/Treatment | Gender specific | LGBTQ+ Lesbian, gay, bisexual, transgender  Asexual  Sexual and gender minorities  Gender dysphoria |
| Tighe 2012 | **Participants:** 644 adolescents and young adults  **Age range:** /  **Mean age:** /  **Distribution/Focus:**  100% of men | **Name:** Alive and Kicking Goals! (AKG) and Project Mentor (PM)  **Setting:** community | Selective | General public information  Awareness and education  Gatekeeper training  Peer-to-peer support | Gender specific | Boy  Men  Male  Hegemonic masculinity  Masculinity strereotypes |

*Notes: DBT: Dialectical behavior therapy*

## Table D5: Studies with gender transformative elements (n = 3)

*The following table details the studies with gender transformative elements, emphasising interventions that not only focus on health outcomes but also actively challenge harmful gender norms.*

| **ID** | **Participants** | **Intervention** | **Preventive approach** | **Strategy/ies** | **Gender approach** | **Vocabulory** |
| --- | --- | --- | --- | --- | --- | --- |
| Duarté-Vélez 2016 | **Participants:** 11 adolescents  **Age range:** 13 - 17  **Mean age:** 15,36  **Distribution:**   - 54,6% of women - 45,4% of men | **Name:** SCBT-SB (socio-cognitive behavioral treatment protocol with suicidal behavior)  **Setting:** medical | Indicated | Management/Treatment | Gender transformative (elements) | Gender Female; male  Girl; boy Gender roles Gender socialisation  Gender perspectives Gender-specific perspectives Heterosexism  Sexual minority |
| Honea 2022 | **Participants:** 50 adolescents and young adults **Age range:** 14 - 24 **Mean age:** /  *In-depth interviews (n = 12)*   - 66,7% of women - 33,3% of men   *Focus groups + interviews (n = 50)*   - 56%% of women - 44% of men | **Name:** Let's Talk **Setting:** community / recruitment in school | Universal | Awareness and education | Gender transformative (elements) | Gender Gender identity  Girl; boy Female; male  Women; men  Gender role (expectations)  Gender expressions  Sexual orientations Transgender; gay; lesbian; queer, transexual identities  Heteronormative masculinity  Masculinity  Binary nature of our current gender system  Non binary people Heteronormative masculinity Sex category |
| Salk 2021 | **Participants:** 148 adolescents and young adults  **Age range:** 18 - 24 **Mean age:** 19,7  **Distribution**  *sex assigned at birth*   - 71% of women - 29% of men   *gender identity*   - 63% identified as women - 23% identified as men - Gender minority patients included 1% of transgender men ; 1% of genderfluid people, 4% of nonbinary people, 1% of other gender identities, 8% of patients who identified with multiple gender identities | **Name:** Intensive Outpatient Program (IOP) named COSTAR (College Option Services for Teens at Risk) (CBT and DBT approaches) **Setting:** school / medical | Indicated | Management/Treatment Contact-information/follow-up care Screening | Gender transformative (elements) | Gender Gender minority Sexual identities  Transgender (male) Female; male Women Genderfluid Non-binary |

*Notes: CBT: Cognitive-behavioral therapy; DBT: Dialectical behavior therapy*

# **Supplementary material E: References of included studies (n = 133)**

*The 133 following references were included in the systematic review.*

Adrian, M., Blossom, J.B., Chu, P.V., Jobes, D., McCauley, E., 2021. Collaborative Assessment and Management of Suicidality for Teens: A Promising Frontline Intervention for Addressing Adolescent Suicidality. Pract Innov (Wash D C) 7, 154–167. <https://doi.org/10.1037/pri0000156>

Adrian, M., McCauley, E., Berk, M.S., Asarnow, J.R., Korslund, K., Avina, C., Gallop, R., Linehan, M.M., 2019. Predictors and moderators of recurring self-harm in adolescents participating in a comparative treatment trial of psychological interventions. J Child Psychol Psychiatry 60, 1123–1132. <https://doi.org/10.1111/jcpp.13099>

Alavi, A., Sharifi, B., Ghanizadeh, A., Dehbozorgi, G., 2013. Effectiveness of cognitive-behavioral therapy in decreasing suicidal ideation and hopelessness of the adolescents with previous suicidal attempts. Iran J Pediatr 23, 467–472.

Allen, J., Rasmus, S.M., Fok, C.C.T., Charles, B., Henry, D., Qungasvik Team, 2018. Multi-Level Cultural Intervention for the Prevention of Suicide and Alcohol Use Risk with Alaska Native Youth: a Nonrandomized Comparison of Treatment Intensity. Prev Sci 19, 174–185. <https://doi.org/10.1007/s11121-017-0798-9>

Anestis, J.C., Charles, N.E., Lee-Rowland, L.M., Barry, C.T., Gratz, K.L., 2020. Implementing Dialectical Behavior Therapy Skills Training With at-Risk Male Youth in a Military-Style Residential Program. Cogn. Behav. Pract. 27, 169–183. <https://doi.org/10.1016/j.cbpra.2019.07.001>

Arenas-Monreal, L., Hidalgo-Solórzano, E., Chong-Escudero, X., Durán-De la Cruz, J.A., González-Cruz, N.L., Pérez-Matus, S., Valdez-Santiago, R., 2022. Suicidal behaviour in adolescents: Educational interventions in Mexico. Health Soc Care Community 30, 998–1005. <https://doi.org/10.1111/hsc.13277>

Asarnow, J.R., Baraff, L.J., Berk, M., Grob, C.S., Devich-Navarro, M., Suddath, R., Piacentini, J.C., Rotheram-Borus, M.J., Cohen, D., Tang, L., 2011. An emergency department intervention for linking pediatric suicidal patients to follow-up mental health treatment. Psychiatr Serv 62, 1303–1309. <https://doi.org/10.1176/ps.62.11.pss6211_1303>

Asarnow, J.R., Berk, M., Hughes, J.L., Anderson, N.L., 2015. The SAFETY Program: a treatment-development trial of a cognitive-behavioral family treatment for adolescent suicide attempters. J Clin Child Adolesc Psychol 44, 194–203. <https://doi.org/10.1080/15374416.2014.940624>

Asarnow, J.R., Hughes, J.L., Babeva, K.N., Sugar, C.A., 2017. Cognitive-Behavioral Family Treatment for Suicide Attempt Prevention: A Randomized Controlled Trial. J Am Acad Child Adolesc Psychiatry 56, 506–514. <https://doi.org/10.1016/j.jaac.2017.03.015>

Aseltine, R.H., DeMartino, R., 2004. An outcome evaluation of the SOS Suicide Prevention Program. Am J Public Health 94, 446–451. <https://doi.org/10.2105/ajph.94.3.446>

Aseltine, R.H., James, A., Schilling, E.A., Glanovsky, J., 2007. Evaluating the SOS suicide prevention program: a replication and extension. BMC Public Health 7, 161. <https://doi.org/10.1186/1471-2458-7-161>

Babeva, K.N., Klomhaus, A.M., Sugar, C.A., Fitzpatrick, O., Asarnow, J.R., 2020. Adolescent Suicide Attempt Prevention: Predictors of Response to a Cognitive-Behavioral Family and Youth Centered Intervention. Suicide Life Threat Behav 50, 56–71. <https://doi.org/10.1111/sltb.12573>

Bailey, E., Alvarez-Jimenez, M., Robinson, J., D’Alfonso, S., Nedeljkovic, M., Davey, C.G., Bendall, S., Gilbertson, T., Phillips, J., Bloom, L., Nicholls, L., Garland, N., Cagliarini, D., Phelan, M., McKechnie, B., Mitchell, J., Cooke, M., Rice, S.M., 2020. An Enhanced Social Networking Intervention for Young People with Active Suicidal Ideation: Safety, Feasibility and Acceptability Outcomes. Int J Environ Res Public Health 17, E2435. <https://doi.org/10.3390/ijerph17072435>

Bailey, E., Robinson, J., Alvarez-Jimenez, M., Nedeljkovic, M., Valentine, L., Bendall, S., D’Alfonso, S., Gilbertson, T., McKechnie, B., Rice, S., 2021. Moderated online social therapy for young people with active suicidal ideation: Qualitative study. J. Med. Internet Res. 23. <https://doi.org/10.2196/24260>

Bailey, E., Spittal, M.J., Pirkis, J., Gould, M., Robinson, J., 2017. Universal Suicide Prevention in Young People. Crisis 38, 300–308. <https://doi.org/10.1027/0227-5910/a000465>

Berk, M.S., Starace, N.K., Black, V.P., Avina, C., 2020. Implementation of Dialectical Behavior Therapy with Suicidal and Self-Harming Adolescents in a Community Clinic. Arch Suicide Res 24, 64–81. <https://doi.org/10.1080/13811118.2018.1509750>

Broadbent, R., Papadopoulos, T., 2014. Improving mental health and wellbeing for young men in the building and construction industry. J Child Adolesc Ment Health 26, 217–227. <https://doi.org/10.2989/17280583.2014.923431>

Busby, D.R., King, C.A., Brent, D., Grupp-Phelan, J., Gould, M., Page, K., Casper, T.C., Pediatric Emergency Care Applied Research Network (PECARN), 2020. Adolescents’ Engagement with Crisis Hotline Risk-management Services: A Report from the Emergency Department Screen for Teen Suicide Risk (ED-STARS) Study. Suicide Life Threat Behav 50, 72–82. <https://doi.org/10.1111/sltb.12558>

Calear, A.L., McCallum, S.M., Christensen, H., Mackinnon, A.J., Nicolopoulos, A., Brewer, J.L., Werner-Seidler, A., Morse, A.R., Kazan, D., Farrer, L.M., Kampel, L., Batterham, P.J., 2022. The Sources of Strength Australia project: A cluster randomised controlled trial of a peer-connectedness school-based program to promote help-seeking in adolescents. J. Affective Disord. 299, 435–443. <https://doi.org/10.1016/j.jad.2021.12.043>

Calear, A.L., Morse, A.R., Batterham, P.J., Forbes, O., Banfield, M., 2021. Silence is Deadly: A controlled trial of a public health intervention to promote help-seeking in adolescent males. Suicide Life Threat Behav 51, 274–288. <https://doi.org/10.1111/sltb.12703>

Choi, H., Park, H., Suarez, M.L., Park, C., Zhao, Z., Wilkie, D.J., 2016. Feasibility of a web-based suicide awareness programme for Asian American college students. BMJ Open 6, e013466. <https://doi.org/10.1136/bmjopen-2016-013466>

Ciffone, J., 2007. Suicide prevention: an analysis and replication of a curriculum-based high school program. Soc Work 52, 41–49. <https://doi.org/10.1093/sw/52.1.41>

Cloutier, P., Gray, C., Sheridan, N., Silverman, A., Cappelli, M., Zemek, R., Jabbour, M., Reid, S., Kennedy, A., 2021. Building Resilience and Attachment in Vulnerable Adolescents (BRAVA): a brief group intervention for adolescents with mild-to-moderate suicidal ideation and their caregivers. Child Adolesc. Ment. Health. <https://doi.org/10.1111/camh.12506>

Cottrell, D.J., Wright-Hughes, A., Collinson, M., Boston, P., Eisler, I., Fortune, S., Graham, E.H., Green, J., House, A.O., Kerfoot, M., Owens, D.W., Saloniki, E.-C., Simic, M., Lambert, F., Rothwell, J., Tubeuf, S., Farrin, A.J., 2018. Effectiveness of systemic family therapy versus treatment as usual for young people after self-harm: a pragmatic, phase 3, multicentre, randomised controlled trial. Lancet Psychiatry 5, 203–216. <https://doi.org/10.1016/S2215-0366(18)30058-0>

Courtney, D.B., Flament, M.F., 2015. Adapted Dialectical Behavior Therapy for Adolescents with Self-injurious Thoughts and Behaviors. J Nerv Ment Dis 203, 537–544. <https://doi.org/10.1097/NMD.0000000000000324>

Cwik, M.F., Tingey, L., Lee, A., Suttle, R., Lake, K., Walkup, J.T., Barlow, A., 2016. Development and piloting of a brief intervention for suicidal American Indian adolescents. Am Indian Alsk Native Ment Health Res 23, 105–124. <https://doi.org/10.5820/aian.2301.2016.105>

Czyz, E.K., King, C.A., Biermann, B.J., 2019. Motivational interviewing-enhanced safety planning for adolescents at high suicide risk: A pilot randomized controlled trial. Journal of Clinical Child and Adolescent Psychology 48, 250–262. <https://doi.org/10.1080/15374416.2018.1496442>

Czyz, E.K., King, C.A., Prouty, D., Micol, V.J., Walton, M., Nahum-Shani, I., 2021. Adaptive intervention for prevention of adolescent suicidal behavior after hospitalization: a pilot sequential multiple assignment randomized trial. J Child Psychol Psychiatry 62, 1019–1031. <https://doi.org/10.1111/jcpp.13383>

Diamond, G.M., Diamond, G.S., Levy, S., Closs, C., Ladipo, T., Siqueland, L., 2012. Attachment-based family therapy for suicidal lesbian, gay, and bisexual adolescents: a treatment development study and open trial with preliminary findings. Psychotherapy (Chic) 49, 62–71. <https://doi.org/10.1037/a0026247>

Diamond, G.S., Wintersteen, M.B., Brown, G.K., Diamond, G.M., Gallop, R., Shelef, K., Levy, S., 2010. Attachment-based family therapy for adolescents with suicidal ideation: a randomized controlled trial. J Am Acad Child Adolesc Psychiatry 49, 122–131. <https://doi.org/10.1097/00004583-201002000-00006>

Dobias, M.L., Schleider, J.L., Jans, L., Fox, K.R., 2021. An online, single-session intervention for adolescent self-injurious thoughts and behaviors: Results from a randomized trial. Behav. Res. Ther. 147. <https://doi.org/10.1016/j.brat.2021.103983>

Donaldson, D., Spirito, A., Esposito-Smythers, C., 2005. Treatment for adolescents following a suicide attempt: results of a pilot trial. J Am Acad Child Adolesc Psychiatry 44, 113–120. <https://doi.org/10.1097/00004583-200502000-00003>

Downs, N., Feng, W., Kirby, B., McGuire, T., Moutier, C., Norcross, W., Norman, M., Young, I., Zisook, S., 2014. Listening to depression and suicide risk in medical students: the Healer Education Assessment and Referral (HEAR) Program. Acad Psychiatry 38, 547–553. <https://doi.org/10.1007/s40596-014-0115-x>

Duarté-Vélez, Y., Torres-Dávila, P., Spirito, A., Polanco, N., Bernal, G., 2016. Development of a treatment protocol for Puerto Rican adolescents with suicidal behaviors. Psychotherapy (Chic) 53, 45–56. <https://doi.org/10.1037/pst0000044>

Eggert, L.L., Thompson, E.A., Randell, B.P., Pike, K.C., 2002. Preliminary effects of brief school-based prevention approaches for reducing youth suicide--risk behaviors, depression, and drug involvement. J Child Adolesc Psychiatr Nurs 15, 48–64. <https://doi.org/10.1111/j.1744-6171.2002.tb00326.x>

Eskin, M., Ertekin, K., Demir, H., 2008. Efficacy of a problem-solving therapy for depression and suicide potential in adolescents and young adults. Cognitive Therapy and Research 32, 227–245. <https://doi.org/10.1007/s10608-007-9172-8>

Esposito-Smythers, C., Spirito, A., Kahler, C.W., Hunt, J., Monti, P., 2011. Treatment of co-occurring substance abuse and suicidality among adolescents: a randomized trial. J Consult Clin Psychol 79, 728–739. <https://doi.org/10.1037/a0026074>

Esposito-Smythers, C., Spirito, A., Uth, R., LaChance, H., 2006. Cognitive behavioral treatment for suicidal alcohol abusing adolescents: development and pilot testing. Am J Addict 15 Suppl 1, 126–130. <https://doi.org/10.1080/10550490601006188>

Fitzpatrick, K.K., Witte, T.K., Schmidt, N.B., 2005. Randomized controlled trial of a brief problem-orientation intervention for suicidal ideation. Behav. Ther. 36, 323–333. <https://doi.org/10.1016/S0005-7894(05)80114-5>

Flynn, A., Zackula, R., Klaus, N.M., McGinness, L., Carr, S., Macaluso, M., 2016. Student Evaluation of the Yellow Ribbon Suicide Prevention Program in Midwest Schools. Prim Care Companion CNS Disord 18. <https://doi.org/10.4088/PCC.15m01852>

Flynn, D., Kells, M., Joyce, M., Corcoran, P., Gillespie, C., Suarez, C., Swales, M., Arensman, E., 2019. Innovations in Practice: Dialectical behaviour therapy for adolescents: multisite implementation and evaluation of a 16-week programme in a public community mental health setting. Child Adolesc. Ment. Health 24, 76–83. <https://doi.org/10.1111/camh.12298>

Freedenthal, S., 2010. Adolescent help-seeking and the Yellow Ribbon Suicide Prevention Program: an evaluation. Suicide Life Threat Behav 40, 628–639. <https://doi.org/10.1521/suli.2010.40.6.628>

Geddes, K., Dziurawiec, S., Lee, C.W., 2013. Dialectical Behaviour Therapy for the Treatment of Emotion Dysregulation and Trauma Symptoms in Self-Injurious and Suicidal Adolescent Females: A Pilot Programme within a Community-Based Child and Adolescent Mental Health Service. Psychiatry J 2013, 145219. <https://doi.org/10.1155/2013/145219>

Gillespie, C., Joyce, M., Flynn, D., Corcoran, P., 2019. Dialectical behaviour therapy for adolescents: a comparison of 16-week and 24-week programmes delivered in a public community setting. Child Adolesc. Ment. Health 24, 266–273. <https://doi.org/10.1111/camh.12325>

Green, J.M., Wood, A.J., Kerfoot, M.J., Trainor, G., Roberts, C., Rothwell, J., Woodham, A., Ayodeji, E., Barrett, B., Byford, S., Harrington, R., 2011. Group therapy for adolescents with repeated self harm: randomised controlled trial with economic evaluation. BMJ 342, d682. <https://doi.org/10.1136/bmj.d682>

Grupp-Phelan, J., Stevens, J., Boyd, S., Cohen, D.M., Ammerman, R.T., Liddy-Hicks, S., Heck, K., Marcus, S.C., Stone, L., Campo, J.V., Bridge, J.A., 2019. Effect of a Motivational Interviewing-Based Intervention on Initiation of Mental Health Treatment and Mental Health After an Emergency Department Visit Among Suicidal Adolescents: A Randomized Clinical Trial. JAMA Netw Open 2, e1917941. <https://doi.org/10.1001/jamanetworkopen.2019.17941>

Gryglewicz, K., Peterson, A., Nam, E., Vance, M.M., Borntrager, L., Karver, M.S., 2021. Caring Transitions - A Care Coordination Intervention to Reduce Suicide Risk Among Youth Discharged From Inpatient Psychiatric Hospitalization. Crisis. <https://doi.org/10.1027/0227-5910/a000795>

Haas, A., Koestner, B., Rosenberg, J., Moore, D., Garlow, S.J., Sedway, J., Nicholas, L., Hendin, H., Mann, J.J., Nemeroff, C.B., 2008. An interactive web-based method of outreach to college students at risk for suicide. J Am Coll Health 57, 15–22. <https://doi.org/10.3200/JACH.57.1.15-22>

Han, J., Batterham, P.J., Calear, A.L., Wu, Y., Xue, J., van Spijker, B.A.J., 2018. Development and pilot evaluation of an online psychoeducational program for suicide prevention among university students: A randomised controlled trial. Internet Interv. 12, 111–120. <https://doi.org/10.1016/j.invent.2017.11.002>

Haruvi Catalan, L., Levis Frenk, M., Adini Spigelman, E., Engelberg, Y., Barzilay, S., Mufson, L., Apter, A., Benaroya Milshtein, N., Fennig, S., Klomek, A.B., 2020. Ultra-Brief Crisis IPT-A Based Intervention for Suicidal Children and Adolescents (IPT-A-SCI) Pilot Study Results. Front. Psychiatry 11. <https://doi.org/10.3389/fpsyt.2020.553422>

Hazell, P.L., Martin, G., Mcgill, K., Kay, T., Wood, A., Trainor, G., Harrington, R., 2009. Group therapy for repeated deliberate self-harm in adolescents: failure of replication of a randomized trial. J Am Acad Child Adolesc Psychiatry 48, 662–670. <https://doi.org/10.1097/CHI.0b013e3181aOacec>

Hetrick, S.E., Yuen, H.P., Bailey, E., Cox, G.R., Templer, K., Rice, S.M., Bendall, S., Robinson, J., 2017. Internet-based cognitive behavioural therapy for young people with suicide-related behaviour (Reframe-IT): a randomised controlled trial. Evid Based Ment Health 20, 76–82. <https://doi.org/10.1136/eb-2017-102719>

Hill, R.M., Dodd, C.G., Gomez, M., Do, C., Kaplow, J.B., 2020. The Safety Planning Assistant: Feasibility and Acceptability of a Web-Based Suicide Safety Planning Tool for At-Risk Adolescents and Their Parents. Evid. Based Pract. Child Adolesc. Mental Heal. 5, 164–172. <https://doi.org/10.1080/23794925.2020.1759469>

Honea, J.C., Keller, S.N., McNeill, V., 2022. Gender differences in youth attitudes towards suicide prevention during a community-based theater program. J. Men’s Health 18. <https://doi.org/10.31083/j.jomh1801020>

Hooven, C., Herting, J.R., Snedker, K.A., 2010. Long-term outcomes for the promoting CARE suicide prevention program. Am J Health Behav 34, 721–736. <https://doi.org/10.5993/ajhb.34.6.8>

Hooven, C., Walsh, E., Pike, K.C., Herting, J.R., 2012. Promoting CARE: including parents in youth suicide prevention. Fam Community Health 35, 225–235. <https://doi.org/10.1097/FCH.0b013e318250bcf9>

Huey, S.J., Henggeler, S.W., Rowland, M.D., Halliday-Boykins, C.A., Cunningham, P.B., Pickrel, S.G., Edwards, J., 2004. Multisystemic therapy effects on attempted suicide by youths presenting psychiatric emergencies. J Am Acad Child Adolesc Psychiatry 43, 183–190. <https://doi.org/10.1097/00004583-200402000-00014>

Humensky, J.L., Coronel, B., Gil, R., Mazzula, S., Lewis-Fernández, R., 2017a. Life is Precious: A Community-Based Program to Reduce Suicidal Behavior in Latina Adolescents. Arch Suicide Res 21, 659–671. <https://doi.org/10.1080/13811118.2016.1242442>

Humensky, J.L., Gil, R.M., Mazzula, S., Diaz, S., Lewis-Fernández, R., 2017b. Life Is Precious: Views of Adolescents and Their Mothers on Methods to Reduce Suicidal Behavior in Latinas. J Sch Nurs 33, 426–433. <https://doi.org/10.1177/1059840517722590>

Hutcherson, K., Kennard, B.D., Michaels, M., Miles, J., 2021. Adapting the SAFETY-Acute Intervention to Improve Quality of Care for Suicidal Youth in Emergency Rooms and Medical Floors. Evid. Based Pract. Child Adolesc. Mental Heal. 6, 369–378. <https://doi.org/10.1080/23794925.2021.1975516>

James, A.C., Winmill, L., Anderson, C., Alfoadari, K., 2011. A Preliminary Study of an Extension of a Community Dialectic Behaviour Therapy (DBT) Programme to Adolescents in the Looked After Care System. Child Adolesc Ment Health 16, 9–13. <https://doi.org/10.1111/j.1475-3588.2010.00571.x>

James, S., Freeman, K.R., Mayo, D., Riggs, M.L., Morgan, J.P., Schaepper, M.A., Montgomery, S.B., 2015. Does Insurance Matter? Implementing Dialectical Behavior Therapy with Two Groups of Youth Engaged in Deliberate Self-harm. Adm Policy Ment Health 42, 449–461. <https://doi.org/10.1007/s10488-014-0588-7>

Jegannathan, B., Dahlblom, K., Kullgren, G., 2014. Outcome of a school-based intervention to promote life-skills among young people in Cambodia. Asian J Psychiatr 9, 78–84. <https://doi.org/10.1016/j.ajp.2014.01.011>

Katz, L.Y., Cox, B.J., Gunasekara, S., Miller, A.L., 2004. Feasibility of dialectical behavior therapy for suicidal adolescent inpatients. J Am Acad Child Adolesc Psychiatry 43, 276–282. <https://doi.org/10.1097/00004583-200403000-00008>

Kemp, K., Webb, M., Wolff, J., Affleck, K., Casamassima, J., Weinstock, L., Spirito, A., 2021. Screening and Brief Intervention for Psychiatric and Suicide Risk in the Juvenile Justice System: Findings from an Open Trial. Evid. Based Pract. Child Adolesc. Mental Heal. 6, 410–419. <https://doi.org/10.1080/23794925.2021.1908190>

Kennard, B., Mayes, T., King, J., Moorehead, A., Wolfe, K., Hughes, J., Castillo, B., Smith, M., Matney, J., Oscarson, B., Stewart, S., Nakonezny, P., Foxwell, A., Emslie, G., 2019. The Development and Feasibility Outcomes of a Youth Suicide Prevention Intensive Outpatient Program. J Adolesc Health 64, 362–369. <https://doi.org/10.1016/j.jadohealth.2018.09.015>

Kennard, B.D., Goldstein, T., Foxwell, A.A., McMakin, D.L., Wolfe, K., Biernesser, C., Moorehead, A., Douaihy, A., Zullo, L., Wentroble, E., Owen, V., Zelazny, J., Iyengar, S., Porta, G., Brent, D., 2018. As Safe as Possible (ASAP): A Brief App-Supported Inpatient Intervention to Prevent Postdischarge Suicidal Behavior in Hospitalized, Suicidal Adolescents. Am J Psychiatry 175, 864–872. <https://doi.org/10.1176/appi.ajp.2018.17101151>

Kim, H.Y., Nam, E.W., Jin, K.N., So, A.Y., 2020. Effectiveness of a school-based mental health education program in an impoverished urban area of Peru. Glob Health Promot 27, 77–86. <https://doi.org/10.1177/1757975918786728>

King, C.A., Arango, A., Kramer, A., Busby, D., Czyz, E., Foster, C.E., Gillespie, B.W., YST Study Team, 2019. Association of the Youth-Nominated Support Team Intervention for Suicidal Adolescents With 11- to 14-Year Mortality Outcomes: Secondary Analysis of a Randomized Clinical Trial. JAMA Psychiatry 76, 492–498. <https://doi.org/10.1001/jamapsychiatry.2018.4358>

King, C.A., Eisenberg, D., Pistorello, J., Coryell, W., Albucher, R.C., Favorite, T., Horwitz, A., Bonar, E.E., Epstein, D., Zheng, K., 2022. Electronic bridge to mental health for college students: A randomized controlled intervention trial. J Consult Clin Psychol 90, 172–183. <https://doi.org/10.1037/ccp0000709>

King, C.A., Eisenberg, D., Zheng, K., Czyz, E., Kramer, A., Horwitz, A., Chermack, S., 2015a. Online suicide risk screening and intervention with college students: a pilot randomized controlled trial. J Consult Clin Psychol 83, 630–636. <https://doi.org/10.1037/a0038805>

King, C.A., Gipson, P.Y., Horwitz, A.G., Opperman, K.J., 2015b. Teen options for change: an intervention for young emergency patients who screen positive for suicide risk. Psychiatr Serv 66, 97–100. <https://doi.org/10.1176/appi.ps.201300347>

King, C.A., Klaus, N., Kramer, A., Venkataraman, S., Quinlan, P., Gillespie, B., 2009. The Youth-Nominated Support Team-Version II for suicidal adolescents: A randomized controlled intervention trial. J Consult Clin Psychol 77, 880–893. <https://doi.org/10.1037/a0016552>

King, C.A., Kramer, A., Preuss, L., Kerr, D.C.R., Weisse, L., Venkataraman, S., 2006. Youth-nominated support team for suicidal adolescents (version 1): A randomized controlled trial. J. Consult. Clin. Psychol. 74, 199–206. <https://doi.org/10.1037/0022-006X.74.1.199>

King, K.A., Strunk, C.M., Sorter, M.T., 2011. Preliminary effectiveness of surviving the teens(®) suicide prevention and depression awareness program on adolescents’ suicidality and self-efficacy in performing help-seeking behaviors. J Sch Health 81, 581–590. <https://doi.org/10.1111/j.1746-1561.2011.00630.x>

King, R., Nurcombe, B., Bickman, L., Hides, L., Reid, W., 2003. Telephone counselling for adolescent suicide prevention: changes in suicidality and mental state from beginning to end of a counselling session. Suicide Life Threat Behav 33, 400–411. <https://doi.org/10.1521/suli.33.4.400.25235>

Klim-Conforti, P., Zaheer, R., Levitt, A.J., Cheung, A.H., Schachar, R., Schaffer, A., Goldstein, B.I., Fefergrad, M., Niederkrotenthaler, T., Sinyor, M., 2021. The impact of a harry potter-based cognitive-behavioral therapy skills curriculum on suicidality and well-being in middle schoolers: A randomized controlled trial. Journal of Affective Disorders 286, 134–141. <https://doi.org/10.1016/j.jad.2021.02.028>

Klimes-Dougan, B., Yuan, C.-, Lee, S., Houri, A.K., 2009. Suicide prevention with adolescents: considering potential benefits and untoward effects of public service announcements. Crisis 30, 128–135. <https://doi.org/10.1027/0227-5910.30.3.128>

Kohls, E., Guenthner, L., Baldofski, S., Eckert, M., Efe, Z., Kuehne, K., Saee, S., Thomas, J., Wundrack, R., Rummel-Kluge, C., 2022. Suicidal Ideation Among Children and Young Adults in a 24/7 Messenger-Based Psychological Chat Counseling Service. Front. Psychiatry 13. <https://doi.org/10.3389/fpsyt.2022.862298>

La Sala, L., Teh, Z., Lamblin, M., Rajaram, G., Rice, S., Hill, N.T.M., Thorn, P., Krysinska, K., Robinson, J., 2021. Can a social media intervention improve online communication about suicide? A feasibility study examining the acceptability and potential impact of the #chatsafe campaign. PLoS One 16, e0253278. <https://doi.org/10.1371/journal.pone.0253278>

Lindow, Janet C., Hughes, J.L., South, C., Gutierrez, L., Bannister, E., Trivedi, M.H., Byerly, M.J., 2020. Feasibility and Acceptability of the Youth Aware of Mental Health (YAM) Intervention in US Adolescents. Arch Suicide Res 24, 269–284. <https://doi.org/10.1080/13811118.2019.1624667>

Lindow, J.C., Hughes, J.L., South, C., Minhajuddin, A., Gutierrez, L., Bannister, E., Trivedi, M.H., Byerly, M.J., 2020. The Youth Aware of Mental Health Intervention: Impact on Help Seeking, Mental Health Knowledge, and Stigma in U.S. Adolescents. J. Adolesc. Health 67, 101–107. <https://doi.org/10.1016/j.jadohealth.2020.01.006>

Manjula, M., Roopesh, B.N., Philip, M., Ravishankar, A., 2018. A brief school based, awareness program for depression and suicidal behaviours in Indian youth. Mental Health and Prevention 11, 47–53. <https://doi.org/10.1016/j.mhp.2018.07.001>

McBee-Strayer, S.M., Thomas, G.V., Bruns, E.M., Heck, K.M., Alexy, E.R., Bridge, J.A., 2019. Innovations in Practice: Intensive crisis intervention for adolescent suicidal ideation and behavior - an open trial. Child Adolesc Ment Health 24, 345–349. <https://doi.org/10.1111/camh.12340>

McGillivray, L., Shand, F., Calear, A.L., Batterham, P.J., Rheinberger, D., Chen, N.A., Burnett, A., Torok, M., 2021. The Youth Aware of Mental Health program in Australian Secondary Schools: 3- and 6-month outcomes. Int. J. Ment. Health Syst. 15. <https://doi.org/10.1186/s13033-021-00503-w>

McManama O’Brien, K.H., LeCloux, M., Ross, A., Gironda, C., Wharff, E.A., 2017. A Pilot Study of the Acceptability and Usability of a Smartphone Application Intervention for Suicidal Adolescents and Their Parents. Arch Suicide Res 21, 254–264. <https://doi.org/10.1080/13811118.2016.1182094>

McManama O’Brien, K.H., Sellers, C.M., Battalen, A.W., Ryan, C.A., Maneta, E.K., Aguinaldo, L.D., White, E., Spirito, A., 2018. Feasibility, acceptability, and preliminary effects of a brief alcohol intervention for suicidal adolescents in inpatient psychiatric treatment. J Subst Abuse Treat 94, 105–112. <https://doi.org/10.1016/j.jsat.2018.08.013>

Mehlum, L., Tørmoen, A.J., Ramberg, M., Haga, E., Diep, L.M., Laberg, S., Larsson, B.S., Stanley, B.H., Miller, A.L., Sund, A.M., Grøholt, B., 2014. Dialectical behavior therapy for adolescents with repeated suicidal and self-harming behavior: a randomized trial. J Am Acad Child Adolesc Psychiatry 53, 1082–1091. <https://doi.org/10.1016/j.jaac.2014.07.003>

Mohatt, G.V., Fok, C.C.T., Henry, D., People Awakening Team, Allen, J., 2014. Feasibility of a community intervention for the prevention of suicide and alcohol abuse with Yup’ik Alaska Native youth: the Elluam Tungiinun and Yupiucimta Asvairtuumallerkaa studies. Am J Community Psychol 54, 153–169. <https://doi.org/10.1007/s10464-014-9646-2>

Muela, A., Balluerka, N., Sansinenea, E., Machimbarrena, J.M., García-Ormaza, J., Ibarretxe, N., Eguren, A., Baigorri, P., 2021. A Social-Emotional Learning Program for Suicide Prevention through Animal-Assisted Intervention. Animals (Basel) 11, 3375. <https://doi.org/10.3390/ani11123375>

Negi, S., Sharma, R., 2020. Effectiveness of healthy emotional expression among girand boys experiencing anxiety and suicide ideation. Med.-Leg. Update 20, 559–565. <https://doi.org/10.37506/mlu.v20i4.1877>

Normand, D., Colin, S., Gaboulaud, V., Baubet, T., Taieb, O., 2018. How to stay in touch with adolescents and young adults after a suicide attempt? Implementation of a 4-phones-calls procedure over 1 year after discharge from hospital, in a Parisian suburb. Encephale 44, 301–307. <https://doi.org/10.1016/j.encep.2017.10.011>

Oldershaw, A., Simic, M., Grima, E., Jollant, F., Richards, C., Taylor, L., Schmidt, U., 2012. The effect of cognitive behavior therapy on decision making in adolescents who self-harm: a pilot study. Suicide Life Threat Behav 42, 255–265. <https://doi.org/10.1111/j.1943-278X.2012.0087.x>

Ougrin, D., Boege, I., Stahl, D., Banarsee, R., Taylor, E., 2013. Randomised controlled trial of therapeutic assessment versus usual assessment in adolescents with self-harm: 2-year follow-up. Arch Dis Child 98, 772–776. <https://doi.org/10.1136/archdischild-2012-303200>

Pasco, S., Wallack, C., Sartin, R.M., Dayton, R., 2012. The impact of experiential exercises on communication and relational skills in a suicide prevention gatekeeper-training program for college resident advisors. J Am Coll Health 60, 134–140. <https://doi.org/10.1080/07448481.2011.623489>

Perera, E.A.R., Kathriarachchi, S.T., 2011. Problem-solving counseling as a therapeutic tool on youth suicidal behavior in the suburban population in Sri Lanka. Indian J Psychiatry 53, 30–35. <https://doi.org/10.4103/0019-5545.75558>

Pickering, T.A., Wyman, P.A., Schmeelk-Cone, K., Hartley, C., Valente, T.W., Pisani, A.R., Rulison, K.L., Brown, C.H., LoMurray, M., 2018. Diffusion of a Peer-Led Suicide Preventive Intervention Through School-Based Student Peer and Adult Networks. Front. Psychiatry 9. <https://doi.org/10.3389/fpsyt.2018.00598>

Pineda, J., Dadds, M.R., 2013. Family intervention for adolescents with suicidal behavior: a randomized controlled trial and mediation analysis. J Am Acad Child Adolesc Psychiatry 52, 851–862. <https://doi.org/10.1016/j.jaac.2013.05.015>

Pisani, A.R., Wyman, P.A., Gurditta, K., Schmeelk-Cone, K., Anderson, C.L., Judd, E., 2018. Mobile Phone Intervention to Reduce Youth Suicide in Rural Communities: Field Test. JMIR Ment Health 5, e10425. <https://doi.org/10.2196/10425>

Pistorello, J., Fruzzetti, A.E., Maclane, C., Gallop, R., Iverson, K.M., 2012. Dialectical behavior therapy (DBT) applied to college students: a randomized clinical trial. J Consult Clin Psychol 80, 982–994. <https://doi.org/10.1037/a0029096>

Portzky, G., van Heeringen, K., 2006. Suicide prevention in adolescents: a controlled study of the effectiveness of a school-based psycho-educational program. J Child Psychol Psychiatry 47, 910–918. <https://doi.org/10.1111/j.1469-7610.2006.01595.x>

Randell, B.P., Eggert, L.L., Pike, K.C., 2001. Immediate post intervention effects of two brief youth suicide prevention interventions. Suicide Life Threat Behav 31, 41–61. <https://doi.org/10.1521/suli.31.1.41.21308>

Rathus, J.H., Miller, A.L., 2002. Dialectical behavior therapy adapted for suicidal adolescents. Suicide Life Threat Behav 32, 146–157. <https://doi.org/10.1521/suli.32.2.146.24399>

Rengasamy, M., Sparks, G., 2019. Reduction of Postdischarge Suicidal Behavior Among Adolescents Through a Telephone-Based Intervention. Psychiatr Serv 70, 545–552. <https://doi.org/10.1176/appi.ps.201800421>

Robinson, J., Hetrick, S., Cox, G., Bendall, S., Yuen, H.P., Yung, A., Pirkis, J., 2016. Can an Internet-based intervention reduce suicidal ideation, depression and hopelessness among secondary school students: results from a pilot study. Early Interv Psychiatry 10, 28–35. <https://doi.org/10.1111/eip.12137>

Robinson, J., Hetrick, S., Cox, G., Bendall, S., Yung, A., Pirkis, J., 2015. The safety and acceptability of delivering an online intervention to secondary students at risk of suicide: findings from a pilot study. Early Interv Psychiatry 9, 498–506. <https://doi.org/10.1111/eip.12136>

Robinson, J., Yuen, H.P., Gook, S., Hughes, A., Cosgrave, E., Killackey, E., Baker, K., Jorm, A., McGorry, P., Yung, A., 2012. Can receipt of a regular postcard reduce suicide-related behaviour in young help seekers? A randomized controlled trial. Early Interv Psychiatry 6, 145–152. <https://doi.org/10.1111/j.1751-7893.2011.00334.x>

Robinson, W.L., Case, M.H., Whipple, C.R., Gooden, A.S., Lopez-Tamayo, R., Lambert, S.F., Jason, L.A., 2016. Culturally Grounded Stress Reduction and Suicide Prevention for African American Adolescents. Pract Innov (Wash D C) 1, 117–128. <https://doi.org/10.1037/pri0000020>

Rossouw, T.I., Fonagy, P., 2012. Mentalization-based treatment for self-harm in adolescents: a randomized controlled trial. J Am Acad Child Adolesc Psychiatry 51, 1304-1313.e3. <https://doi.org/10.1016/j.jaac.2012.09.018>

Rotheram-Borus, M.J., Piacentini, J., Cantwell, C., Belin, T.R., Song, J., 2000. The 18-month impact of an emergency room intervention for adolescent female suicide attempters. J Consult Clin Psychol 68, 1081–1093. [https://doi.org/10.1037//0022-006x.68.6.1081](https://doi.org/10.1037/0022-006x.68.6.1081)

Russon, J., Morrissey, J., Dellinger, J., Jin, B., Diamond, G., 2021. Implementing Attachment-Based Family Therapy for Depressed and Suicidal Adolescents and Young Adults in LGBTQ+ Services. Crisis 43, 500–507. <https://doi.org/10.1027/0227-5910/a000821>

Salk, R.H., Moses-Kolko, E.L., Chugani, C.D., Mastruserio, S., Wentroble, E., Blackburn, V., Poling, K., Sakolsky, D., Brent, D., Goldstein, T.R., 2021. An intensive outpatient program for suicidal college students. J Am Coll Health 1–6. <https://doi.org/10.1080/07448481.2021.1879814>

Samuolis, J., Harrison, A.J., Flanagan, K., 2020. Evaluation of a Peer-Led Implementation of a Suicide Prevention Gatekeeper Training Program for College Students. Crisis 41, 331–336. <https://doi.org/10.1027/0227-5910/a000638>

Schiffler, T., Seiler-Ramadas, R., Štefanac, S., Haider, S., Mües, H.M., Grabovac, I., 2022. Implementation of a Mobile DBT App and Its Impact on Suicidality in Transitional Age Youth with Borderline Personality Disorder: A Qualitative Study. Int. J. Environ. Res. Public Health 19. <https://doi.org/10.3390/ijerph19020701>

Silk, K.J., Perrault, E.K., Nazione, S.A., Pace, K., Collins-Eaglin, J., 2017. Evaluation of a Social Norms Approach to a Suicide Prevention Campaign. J Health Commun 22, 135–142. <https://doi.org/10.1080/10810730.2016.1258742>

Silverstone, P.H., Bercov, M., Suen, V.Y.M., Allen, A., Cribben, I., Goodrick, J., Henry, S., Pryce, C., Langstraat, P., Rittenbach, K., Chakraborty, S., Engels, R.C., McCabe, C., 2015. Initial Findings from a Novel School-Based Program, EMPATHY, Which May Help Reduce Depression and Suicidality in Youth. PLoS One 10, e0125527. <https://doi.org/10.1371/journal.pone.0125527>

Silverstone, P.H., Bercov, M., Suen, V.Y.M., Allen, A., Cribben, I., Goodrick, J., Henry, S., Pryce, C., Langstraat, P., Rittenbach, K., Chakraborty, S., Engles, R.C., McCabe, C., 2017. Long-term Results from the Empowering a Multimodal Pathway Toward Healthy Youth Program, a Multimodal School-Based Approach, Show Marked Reductions in Suicidality, Depression, and Anxiety in 6,227 Students in Grades 6-12 (Aged 11-18). Front Psychiatry 8, 81. <https://doi.org/10.3389/fpsyt.2017.00081>

Spirito, A., Boergers, J., Donaldson, D., Bishop, D., Lewander, W., 2002. An intervention trial to improve adherence to community treatment by adolescents after a suicide attempt. J Am Acad Child Adolesc Psychiatry 41, 435–442. <https://doi.org/10.1097/00004583-200204000-00016>

Spirito, A., Wolff, J.C., Seaboyer, L.M., Hunt, J., Esposito-Smythers, C., Nugent, N., Zlotnick, C., Miller, I., 2015. Concurrent treatment for adolescent and parent depressed mood and suicidality: feasibility, acceptability, and preliminary findings. J Child Adolesc Psychopharmacol 25, 131–139. <https://doi.org/10.1089/cap.2013.0130>

Tang, T.-C., Jou, S.-H., Ko, C.-H., Huang, S.-Y., Yen, C.-F., 2009. Randomized study of school-based intensive interpersonal psychotherapy for depressed adolescents with suicidal risk and parasuicide behaviors. Psychiatry Clin Neurosci 63, 463–470. <https://doi.org/10.1111/j.1440-1819.2009.01991.x>

Taylor-Rodgers, E., Batterham, P.J., 2014. Evaluation of an online psychoeducation intervention to promote mental health help seeking attitudes and intentions among young adults: randomised controlled trial. J Affect Disord 168, 65–71. <https://doi.org/10.1016/j.jad.2014.06.047>

Thompson, E.A., Eggert, L.L., Randell, B.P., Pike, K.C., 2001. Evaluation of indicated suicide risk prevention approaches for potential high school dropouts. Am J Public Health 91, 742–752. <https://doi.org/10.2105/ajph.91.5.742>

Tighe, J., McKay, K., 2012. Alive and Kicking Goals!: Preliminary findings from a Kimberley suicide prevention program. Advances in Mental Health 10, 240–245. <https://doi.org/10.5172/jamh.2012.10.3.240>

Trinh, T., Goebert, D., 2020. Youth Voice in Suicide Prevention in Hawai’i. Hawaii J Health Soc Welf 79, 71–75.

Wasserman, D., Hoven, C.W., Wasserman, C., Wall, M., Eisenberg, R., Hadlaczky, G., Kelleher, I., Sarchiapone, M., Apter, A., Balazs, J., Bobes, J., Brunner, R., Corcoran, P., Cosman, D., Guillemin, F., Haring, C., Iosue, M., Kaess, M., Kahn, J.-P., Keeley, H., Musa, G.J., Nemes, B., Postuvan, V., Saiz, P., Reiter-Theil, S., Varnik, A., Varnik, P., Carli, V., 2015. School-based suicide prevention programmes: the SEYLE cluster-randomised, controlled trial. Lancet 385, 1536–1544. <https://doi.org/10.1016/S0140-6736(14)61213-7>

Wharff, E.A., Ginnis, K.B., Ross, A.M., White, E.M., White, M.T., Forbes, P.W., 2019. Family-Based Crisis Intervention With Suicidal Adolescents: A Randomized Clinical Trial. Pediatr Emerg Care 35, 170–175. <https://doi.org/10.1097/PEC.0000000000001076>

Wharff, E.A., Ginnis, K.M., Ross, A.M., 2012. Family-based crisis intervention with suicidal adolescents in the emergency room: a pilot study. Soc Work 57, 133–143. <https://doi.org/10.1093/sw/sws017>

Wijana, M.B., Enebrink, P., Liljedahl, S.I., Ghaderi, A., 2018. Preliminary evaluation of an intensive integrated individual and family therapy model for self-harming adolescents. BMC Psychiatry 18. <https://doi.org/10.1186/s12888-018-1947-9>

Wilcox, H.C., Kellam, S.G., Brown, C.H., Poduska, J.M., Ialongo, N.S., Wang, W., Anthony, J.C., 2008. The impact of two universal randomized first- and second-grade classroom interventions on young adult suicide ideation and attempts. Drug Alcohol Depend 95 Suppl 1, S60-73. <https://doi.org/10.1016/j.drugalcdep.2008.01.005>

Wood, A., Trainor, G., Rothwell, J., Moore, A., Harrington, R., 2001. Randomized trial of group therapy for repeated deliberate self-harm in adolescents. J Am Acad Child Adolesc Psychiatry 40, 1246–1253. <https://doi.org/10.1097/00004583-200111000-00003>

Woodberry, K.A., Popenoe, E.J., 2008. Implementing Dialectical Behavior Therapy With Adolescents and Their Families in a Community Outpatient Clinic. Cogn. Behav. Pract. 15, 277–286. <https://doi.org/10.1016/j.cbpra.2007.08.004>

Wyman, P.A., Brown, C.H., LoMurray, M., Schmeelk-Cone, K., Petrova, M., Yu, Q., Walsh, E., Tu, X., Wang, W., 2010. An outcome evaluation of the Sources of Strength suicide prevention program delivered by adolescent peer leaders in high schools. Am J Public Health 100, 1653–1661. <https://doi.org/10.2105/AJPH.2009.190025>

Xavier, A., Otero, P., Blanco, V., Vázquez, F.L., 2019. Efficacy of a problem-solving intervention for the indicated prevention of suicidal risk in young Brazilians: Randomized controlled trial. Suicide Life Threat Behav 49, 1746–1761. <https://doi.org/10.1111/sltb.12568>
